# Pseudovibriamide B from marine sponge-associated bacteria acts as a selective antibiotic antidote

**DOI:** 10.1093/ismeco/ycag014

**Published:** 2026-01-22

**Authors:** Vitor Lourenzon, Yitao Dai, Mandisa Timba, Subhash Yadav, Gabrielle Mingolelli, Matthew Henke, Detmer Sipkema, Alessandra S Eustáquio

**Affiliations:** Department of Pharmaceutical Sciences and Center for Biomolecular Sciences, Retzky College of Pharmacy, University of Illinois Chicago, Chicago, IL, 60607, United States; Department of Pharmaceutical Sciences and Center for Biomolecular Sciences, Retzky College of Pharmacy, University of Illinois Chicago, Chicago, IL, 60607, United States; Department of Pharmaceutical Sciences and Center for Biomolecular Sciences, Retzky College of Pharmacy, University of Illinois Chicago, Chicago, IL, 60607, United States; Laboratory of Microbiology, Wageningen University, P.O. Box 8033, 6700 EH Wageningen, the Netherlands; Department of Pharmaceutical Sciences and Center for Biomolecular Sciences, Retzky College of Pharmacy, University of Illinois Chicago, Chicago, IL, 60607, United States; Department of Pharmaceutical Sciences and Center for Biomolecular Sciences, Retzky College of Pharmacy, University of Illinois Chicago, Chicago, IL, 60607, United States; Laboratory of Microbiology, Wageningen University, P.O. Box 8033, 6700 EH Wageningen, the Netherlands; Department of Pharmaceutical Sciences and Center for Biomolecular Sciences, Retzky College of Pharmacy, University of Illinois Chicago, Chicago, IL, 60607, United States

**Keywords:** *Pseudovibrio*, marine bacteria, marine sponges, antibiotic antidote

## Abstract

Broad-spectrum antibiotics, while effective against pathogens, can disrupt microbiota leading to dysbiosis. In natural systems, where most antibiotic classes were first identified, some collateral damage control may have evolved as a microbiota protection strategy to the production of broad-spectrum antibiotics. In this work, we investigated whether pseudovibriamide B (PB), a depsipeptide produced by the marine sponge isolated bacterium *Pseudovibrio brasiliensis* Ab134, can function as a selective antidote to the commercial broad-spectrum antibiotic blasticidin S. An analogue of blasticidin S, named P10, was previously isolated from marine sponges. To improve access to PB and enable testing of the hypothesis, we developed an overexpression strategy targeting core biosynthetic genes, resulting in a more than three-fold increase in PB production. Additionally, we established an assay for antidote testing, the Minimum Antidote Concentration (MAC) assay, which enabled robust identification of antidote activity and quantification of the minimum concentration required to rescue a strain at a given antibiotic dose. The MAC assay revealed that PB selectively protects *Bacillus cereus* and a marine sponge-associated *Bacillus* sp., but not pathogens or other sponge-associated isolates, indicating narrow-spectrum antidote activity. These findings support a role of pseudovibriamides as selective antibiotic antagonists and provide a framework for future work of natural antidotes for targeted microbiota protection both in ecological and clinical settings.

## Introduction

Broad-spectrum antibiotics are widely used to treat human infections [1]. It is now recognized that disruption of the human microbiome by broad-spectrum antibiotic treatment can increase susceptibility to or worsen the outcome of disease [2–7]. In a recent study, the Typas lab provided proof of concept for selective antibiotic antidotes as an approach to avoid collateral damage by broad-spectrum antibiotics [8]. The authors identified four drugs from the Prestwick Chemical Library that selectively protected abundant gut commensals from two broad-spectrum antibiotics (erythromycin and doxycycline) but that did not affect the antibiotics’ efficacy against pathogens.

Most clinically relevant antibiotic classes are natural products derived from bacteria (65%) or fungi (15%) with the remainder being synthetic (19%) [9]. Given that many natural antibiotics are broad spectrum, the same challenge of preventing collateral damage to beneficial microbes within microbial communities is likely encountered in nature in terrestrial and marine environments such as in soil, plants and animals. Indeed, the antibiotic antidote approach is a solution that appears to have evolved in nature.

**Figure 1:**
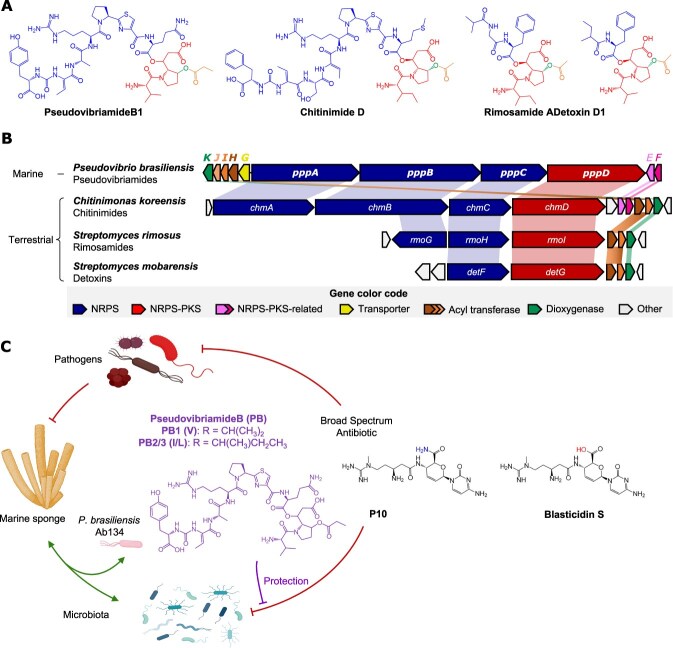
Bacterial antibiotic antidotes. (A) Representative structures color-coded according to the encoding genes. (B) Biosynthetic gene clusters. NRPS, nonribosomal peptide synthetase. PKS, polyketide synthase. (C) Hypothesis tested in this study. The marine sponge isolate *P. Brasiliensis* produces two major PB analogues, PB1 containing a valine (V) residue and PB2/3 containing isoleucine/leucine (I/L) residues. The broad-spectrum antibiotic P10 (previously isolated from sponge samples), may be damaging to the sponge microbiota. We hypothesized that pseudovibriamides may selectively protect some of the sponge microbiota but not pathogens. Blasticidin S is a commercially available analog of P10 used in this study to test the hypothesis.

Detoxins were the first natural antibiotic antidotes to be discovered and shown to function as selective antidotes to blasticidin S, a broad-spectrum, peptidyl nucleoside antibiotic used as a fungicide to treat rice blast disease [10]. Detoxin D1 (Fig. 1A), as the most active derivative, protects some microbes such as *Bacillus cereus*, and it also protects the rice plant, but does not protect *E. coli*, *Pseudomonas fluorescens* or *Piricularia oryzae*, the fungal pathogen that causes rice blast [10, 11]. Studies with detoxin D1 suggested a mechanism in which the antidote causes efflux of blasticidin S from cells [12]. Rimosamide A and chitinimide D (Fig. 1A) were later discovered via genome mining and shown to have antidote activity in the *B. cereus*-blasticidin S assay [13, 14]. According to studies with detoxins, the full-length depsipeptide products are the most active, with the hybrid peptide-polyketide precursor (red genes in Fig. 1B and red structural component in Fig. 1A) showing nearly three orders of magnitude reduced activity [15].

We have recently discovered pseudovibriamide B (PB) from marine *Pseudovibrio brasiliensis* Ab134, the structure of which resembles detoxin D1, rimosamide A and chitinimide D from soil bacteria (Fig. 1A) [16]. *Pseudovibrio* α-Proteobacteria have been isolated predominantly from marine sponges but also from other marine organisms such as corals, tunicates, flatworms and algae [17]. We previously showed that pseudovibriamide biosynthesis is encoded in the *ppp* biosynthetic gene cluster (BGC), which is found in around two-thirds of sequenced *Pseudovibrio* strains isolated from diverse marine sponges and a tunicate [16]. Additional work showed that pseudovibriamides promote flagella-mediated motility by modulating gene transcription in *P. brasiliensis* Ab134 [18].

Blasticidin S was first isolated from *Streptomyces* soil bacteria [19], but an analogue called P10 (Fig. 1C) was recently isolated from marine sponges [20]. *Pseudovibrio* itself has been proposed to contribute to marine sponge health [17]. Marine sponges are ancient animals that form important symbiotic relationships with their microbes [21]. Thus, broad-spectrum antibiotics known to be produced in sponges could cause dysbiosis in the sponge animal, unless there was a strategy in place to prevent dysbiosis.

Here we tested the hypothesis that PB may act as a selective antidote to protect marine sponge bacteria from broad-spectrum antibiotics without protecting pathogens (Fig. 1C). We first tested the antidote hypothesis in a *B. cereus*-blasticidin S assay using extract fractions enriched in PB. We then attempted three strategies to improve production and facilitate wider testing and were able to improve PB production by overexpressing genes that were limiting its biosynthesis. A minimum antidote concentration assay showed that PB has a narrow spectrum of activity, protecting a *Bacillus* sp. marine sponge isolate in addition to *B. cereus* but showing no activity against a sponge and a coral pathogen or other sponge isolates tested.

## Materials and methods

### Bacterial strains and general cultivation conditions

The source of bacterial strains used in this study can be found in Table S1. *P. brasiliensis* Ab134 [16] was cultivated at 30°C in BD Difco Marine Broth 2216 (MB) or on BD Difco Marine Agar 2216 (MA). *E. coli* strains were cultured at 37°C in BD Difco Luria Broth (LB), BD Difco LB agar, or terrific broth (24 g yeast extract (BD Bacto™), 12 g tryptone (Fisher Bioreagents), 9.4 g potassium phosphate dibasic (Sigma-Aldrich), and 2.2 g potassium phosphate monobasic (Fisher Scientific) per 1 L Milli-Q water). Marine isolates were cultivated at 30°C in MB. *Burkholderia* sp. FERM BP-3421 Δ*fr9A* [22] was cultured in LB, LB agar, or 2S4G medium (40 g glycerol (Fisher Scientific), 20 g Hy-Soy peptone (Kerry Bioscience), 2 g ammonium sulfate (Sigma-Aldrich), 0.1 g magnesium sulfate heptahydrate (Sigma-Aldrich), and 2 g calcium carbonate (Acros Organics) per 1 L Milli-Q water). *B. cereus* NRRL B-3711 was cultured at 30°C in MB. Cultures were incubated for ~20 hours, unless otherwise stated. Media, antibiotics, and promoter inducers used for each strain are detailed in Table S2.

### Disk diffusion antidote assay

A single colony of *B. cereus* NRRL B-3711 was picked to start an overnight culture in a 12 ml culture tube. The next day, 150 μl of the overnight culture with an optical density absorbance at 600 nm (OD_600_) ~ 1.0 was spread evenly on MA using a cotton swab. While the plates were drying, blank sterile test disks (6 mm, VWR 470178–408) were loaded with either 10 μl of 1 mg/ml blasticidin S in water, or solid-phase extraction fractions in methanol. The loaded disks were allowed to dry in the biological safety cabinet for at least an hour. Once both disks and plates were completely dry, the disks were placed on the agar using forceps. Plates were sealed and incubated at 37°C.

### Spent media antidote assay

**Figure 2:**
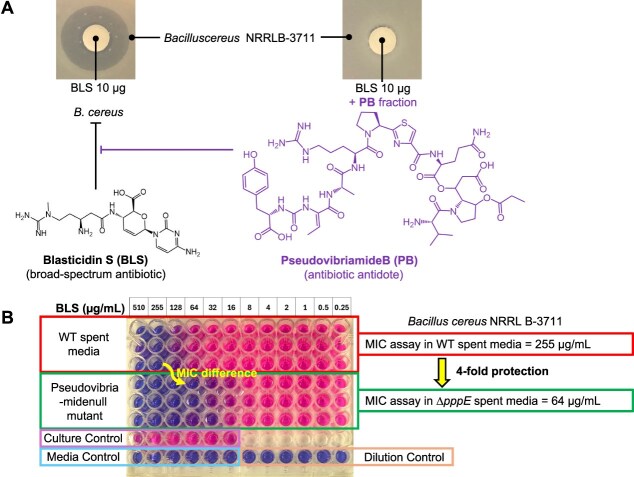
Initial antibiotic antidote assays. (A) Disk diffusion assay performed in 20 ml marine agar plates. (B) Enriched spent media minimal inhibitory concentration (MIC) comparison. The MIC assay uses resazurin dye to detect bacterial growth. If bacteria growth is inhibited, resazurin remains blue (oxidized form). Metabolically active cells reduce resazurin to a pink form.

*P. brasiliensis* Ab134 wild-type (WT) and pseudovibriamide-defective Δ*pppE* strains [18] were cultured in 20 ml of MB at 30°C, 200 rpm, for 3 days. The cultures were sonicated for 30 min and filtered through a 0.22 μm vacuum filtration bottle to obtain sterile spent medium from each culture. The spent media were then enriched (19:1) with a solution containing 100 g of peptone and 20 g of yeast extract per liter of water to recapitulate the carbon and nitrogen sources of MB (Fig. S1). To assess the antidote activity of pseudovibriamides produced by the WT strain, two minimum inhibitory concentration (MIC) assays were performed on the same 96-well plate: one in enriched spent medium from *P. brasiliensis* WT (rows A to C), and one in enriched spent medium from *P. brasiliensis* Δ*pppE* (rows D to E). The MIC plate was incubated for 48 hours at 30°C. Afterwards, 30 μl of a 0.015% resazurin solution was added to each well, followed by 3 hours of incubation at 30°C. In the presence of viable cells, resazurin (a blue dye) is reduced to resorufin, a pink, fluorescent dye [23] (Fig. 2). After 3 hours of incubation at 30°C, fluorescence was measured using an Infinite M Plex plate reader (Tecan) with excitation at 560 nm and emission at 590 nm. Additionally, a picture of each plate was taken (Fig. S1).

### Plasmid construction

Ab134 genomic DNA isolated from a MB overnight culture using the GenElute™ Bacterial Genomic DNA Kit (Sigma-Aldrich) was used as template for amplification of genes (construction of pVLDEF, pVLHIJK, and pMTX01), and for the amplification of homology arms (construction of pMTD01). Q5® High-Fidelity DNA Polymerase (New England Biolabs) was used for 30 cycles PCR amplification according to the manufacturer’s instructions and using the primers and annealing temperatures listed in Table S3 at 500 nM each primer. After restriction digests with the appropriate enzymes (Table S4) if applicable, T4 DNA ligase (Thermo Scientific) was used to build pVLHIJK and pMTX01. NEBuilder® HiFi DNA Assembly Master Mix was used to build pVL001, pVLDEF, and pMTD01. Plasmids were constructed using *E. coli* S17–1 (pVL001, pVLDEF, pVLHIJK, and pMTX01) or *E. coli* S17–1 λ*pir* (pMTD01). Ligation or assembly mixtures were transferred into *E. coli* by chemical transformation. The obtained plasmids were first confirmed by plasmid isolation using ZR Plasmid Miniprep Classic kit (Zymo Research), restriction digestion and gel electrophoresis, followed by whole plasmid sequencing (Plasmidsaurus). A list of vectors and constructed plasmids is presented in Table S4. Further details on each plasmid construction, plasmid transfer into bacterial strains and in-frame deletion of *pppO* can be found under Supplementary Methods and Table S5.

### Production cultures

All *P. brasiliensis* strains, and the heterologous hosts were cultured in 250 ml Erlenmeyer with 20 ml of the appropriate production media and supplemented with the required antibiotics and inducers (Table S2). The large-scale cultivation of *P. brasiliensis* Ab134 pVLDEF was performed in six 2.8 L Fernbach flasks at a time, each containing 500 ml of MB supplemented with tetracycline (100 μg/ml), and the two promoter inducers l-arabinose (100 mM) and l-rhamnose (50 mM). The cultures were incubated at 30°C and 200 rpm for 3 days. Cultivation was repeated 4 times, totaling 12 L of culture.

### Culture extraction

All cultures were extracted with 5% of conditioned XAD 7-HP resin (1 g wet resin per 20 ml cultures and 25 g for each 500 ml culture). The resin was added to each culture flask at the end of cultivation and flasks were shaken at 200 rpm for 1 hour. The resin and cells were filtered in a Büchner funnel under vacuum. The resin-cell mixture was soaked in methanol (half of culture volume) and agitated for one hour. The methanol extract was then filtered through gravity. Methanol extraction of the resin-cell mixture was repeated two more times to ensure maximum extraction. The three combined extracts were dried under reduced pressure.

### Metabolite analysis and isolation

Analyses of pseudovibriamide production were performed by LC–MS as described in the Supplementary Methods. Pseudovibriamide B was isolated from 12 L (16.7 g crude extract) of *P. brasiliensis* pVLDEF culture using flash, size exclusion and high-performance liquid chromatography yielding pseudovibriamide B1 (3 mg) and pseudovibriamide B2 and B3 (3.4 mg) as described in the Supplementary Methods (Supplementary Methods, Fig. S2 – S9).

### Growth curves

Seed cultures were prepared by inoculating 10 μl of cryo-preserved cultures into 5 ml of MB and incubating at 30°C, 200 rpm, overnight. A second overnight culture was prepared by inoculating 50 μl of the first seed culture into 5 ml of MB and incubating at 30°C, 200 rpm. Standardized cultures to OD_600_ = 0.01 (100 μl each well, 4 replicates for each strain) were aliquoted into 96-well plates (Falcon 351 172, flat bottom, non-treated, clear plates) using a multi-channel pipettor. Four wells were filled with 100 μl of MB (blank). An Infinite M Plex plate reader (Tecan) was used. Plates were incubated at 30°C for 24 or 72 hours. Every 30 minutes the plates were first shaken for 30 seconds (linear shaking with 1 mm of amplitude and 887 rpm) followed by an OD_600_ reading.

### Strain authentication

The eight marine sponge isolates received from the Sipkema lab were authenticated by 16S rRNA gene sequencing. The strains were streaked onto a MA plate, and single colonies were collected for colony PCR and to start a 5 ml MB culture. The single colonies were collected with a sterile toothpick and resuspended into 10 μl of water. The cell suspension was used as DNA source for colony PCR with Q5® High-Fidelity DNA Polymerase (New England Biolabs) for 30 cycles according to the manufacturer’s instructions and using the primers and annealing temperature listed in Table S3 at 500 nM each primer. The PCR products were purified with DNA Clean & Concentrator-5 kit (Zymo, D40**1**4), followed by sequencing (Plasmidsaurus). The 16S sequences obtained were analyzed using BLAST to confirm the reported strain taxonomy (Table S6).

### MIC assay

The protocol was adapted from [24] with modifications. Two-stage seed cultures were prepared as described above for the growth curves. 50 μl of the second seed cultures were used to start fresh cultures in 5 ml of MB that were incubated at 30°C until OD_600_ 0.1, then diluted 1:20 in fresh media. The assays were performed in MB and incubated for 48 hours at 30°C. The MIC assays were revealed with a resazurin cell-viability assay, as previously described [23].

### Minimum antidote concentration assay

The minimum antidote concentration (MAC) assay was developed by adapting a MIC assay. In the MAC assay, a solution A containing a concentration 1.25–2× higher than the MIC observed for the antibiotic and bacteria to be tested was added in all the wells (rows A to C). Afterwards, solution B was prepared by diluting the antidote with solution A. *B. cereus* was tested with a starting concentration of 100 μg/ml of Pmix. For all other strains, if no protection was initially observed, the starting Pmix concentration was increased to 512 μg/ml. Antidote solution B was added to the first well of the row and a serial dilution was performed to obtain 12 concentrations of the antidote, all with the same concentration of antibiotic. In addition, several controls were added to the *B. cereus* plate: dilution of the antidote without the antibiotic (row D), bacterial culture without antibiotic (F1-F3), media sterility control (F4-F6), bacterial culture in solution A (G1-G3), solution A sterility control (G4-G6), and serial dilution sterility control (H9-H12). Moreover, a positive control was added to each plate when screening new bacteria (*B. cereus* in blasticidin S 175 μg/ml with a serial dilution of Pmix, row E). Other *B. cereus* controls included were *B. cereus* culture without antibiotic (F7-F9), media sterility control (F10-F12), *B. cereus* culture in solution A (G7-G9), and solution A sterility control (G10-G2). The plates were incubated for 48 hours at 30°C, and then a resazurin cell-viability assay was performed as previously described [23].

## Results

### Pseudovibriamide B acts as a blasticidin S antibiotic antidote with *Bacillus cereus* as test strain

To explore the potential antibiotic-antidote activity reported for related natural products (Fig. 1A), we first tested PB using a disk diffusion assay (Fig. 2A). The disk diffusion assay showed that 10 μg of commercial blasticidin S produced a visible and homogeneous inhibition zone against *Bacillus cereus* NRRL B-3711. Because pure PB was not available, we used culture extract fractions. The fraction containing PB was able to rescue the blasticidin S inhibition of *B. cereus* completely (Fig. 2A).

Due to the low titers and difficulties in isolating PB, we next developed an assay to screen for antidote activity using the spent medium (supernatant) of *P. brasiliensis* Ab134 cultures enriched with marine broth carbon and nitrogen sources. As a negative control, we used the enriched spent medium from the knockout mutant *P. brasiliensis* Ab134 ∆*pppE*, which does not produce any pseudovibriamides (Table S7). The assay was designed as a MIC test using enriched culture supernatants as the growth medium (Fig. S1). This setup allowed the test bacterium to grow in the presence (wild-type supernatant) or absence (∆*pppE* supernatant) of pseudovibriamides, while exposed to a serial dilution of blasticidin S. If the compounds in the wild-type supernatant conferred protection, the MIC of blasticidin S would be higher than that observed with the ∆*pppE* supernatant.

To validate the supernatant antidote MIC assay, we tested it with *B. cereus* NRRL B-3711. The initial concentration of blasticidin S was 510 μg/ml, and it was serially diluted to 0.25 μg/ml. The MIC of *B. cereus* NRRL B-3711 in the wild-type supernatant was 255 μg/ml, four times higher than the MIC observed in the ∆*pppE* supernatant. These results demonstrated that the presence of pseudovibriamides, even at low concentrations in the supernatant, can protect *B. cereus* NRRL B-3711 against blasticidin S (Fig. 2B).

### Overexpression of *pppDEF* improved PB production

While the enriched spent medium assay was useful for initial testing, we next directed our efforts to isolating pseudovibriamides to obtain minimal antidote concentrations. However, pseudovibriamides are produced in low yields, and the previously reported process for obtaining pure compounds relies on semi-solid media cultures and complex extraction and purification methods [16]. Therefore, we aimed to improve access to pseudovibriamides by engineering their biosynthesis and optimizing the culture, extraction, and purification methods.

We first investigated a two-component system (TCS) located ~3 kb upstream of the BGC. The TCS was deleted and overexpressed to test its potential involvement in activating or repressing the *ppp* BGC. However, neither approach improved pseudovibriamide production (Supplementary Results, SI Fig. S10).

**Figure 3:**
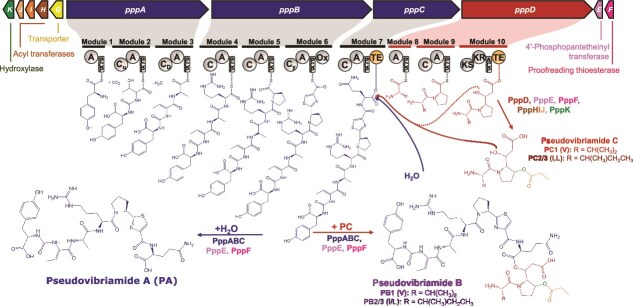
Proposed biosynthesis of pseudovibriamides. The heptapeptide (blue) is assembled by the nonribosomal peptide synthetase (NRPS) complex PppABC, at the end of which it is transferred to the TE in module 7. Nucleophilic attack of the ester bond with water (hydrolysis) results in pseudovibriamide A (PA). Alternatively, nucleophilic attack of the ester bond with the hydroxyl group of pre-PC (red, product of PppD before tailoring by PppHIJK) or PC (red/green/orange) results in PB. PC can be detected by mass spectrometry, but has not been isolated [18].

With the knockout library previously generated [18], we were able to determine which genes were required for the biosynthesis of each structural feature of the pseudovibriamides (Table S7). Based on this knowledge, we first designed a heterologous expression system for the genes responsible for the biosynthesis of pseudovibriamide C (PC). PC is a precursor to PB, requiring the smallest cloned region (Fig. 3). If heterologous expression was successful with PC, we would then add genes *pppABC* to complete the biosynthesis of PB.

Two plasmids were designed to clone the genes required for the biosynthesis of PC, pVLDEF, containing the core genes *pppDEF*, and pVLHIJK, containing the accessory genes *pppHIJK*. The two plasmids were introduced into two heterologous hosts, *E. coli* BL21, a model γ-proteobacterium commonly used for heterologous production of proteins and natural products, and *Burkholderia* sp. FERM BP-3421 ∆*fr9A*, a β-proteobacterium under development in our laboratory. Both heterologous hosts were cultured under multiple culture conditions; however, PC was not detected in any of the tested cultures (Figs. S11–S13). An α-proteobacterial host, matching the taxonomic class of *Pseudovibrio*, may have fared better, but because we did not have immediate access to one, we decided to test an alternative strategy instead.

Without a functioning heterologous expression system, our efforts were redirected toward the native producer strain, seeking ways to engineer *P. brasiliensis* Ab134 to improve the production of PB. Two hypotheses are conceivable to explain the observation of the full-length product PB and of the precursors PA and PC in culture extracts (Fig. 3). PB could be initially the main product of the *ppp* BGC and the ester bond would be later hydrolyzed into PA and PC. None of the *ppp* genes appear to function as an esterase to support this hypothesis [18], although an esterase could still be present outside of the *ppp* cluster. The nonribosomal peptide synthetase–polyketide synthase (NRPS–PKS) modules contain two distinct thioesterase (TE) domains, one at the end of module 7 and one at the end of module 10. Consistent with this architecture, an alternative hypothesis is that the heptapeptide could be released from the TE domain on module 7 either through hydrolysis by a water molecule, resulting in PA, or by nucleophilic attack by the hydroxyl group present on PC or pre-PC (product of PppD without tailoring by PppHIJK), resulting in the formation of PB (Fig. 3). If the latter hypothesis is true, increasing the availability of PC/pre-PC should improve production of PB.

**Figure 4:**
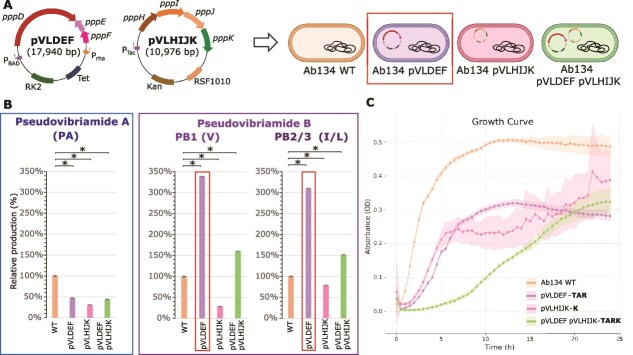
Overexpression of *pppDEF* improved PB production in *P. Brasiliensis* Ab134. (A) Plasmid design for genes related to PC biosynthesis. Plasmids are not scaled to size. (B) Comparative quantification of the production of pseudovibriamides A and B in different strains of *P. brasiliensis* Ab134. The area under the curve (AUC) of mass spectrometry extracted features for each compound in the wild type was set as 100%, and the AUC in the plasmid containing strains are represented relative to the wild type. ^*^P-value <0.05. (C) Growth curves of the four strains depicted in panel A measured based on optical density at 600 nm (OD). Bold letters in front of plasmid names represent the antibiotics and promoter inducers used for each culture: Tetracycline (T), kanamycin (K), l-Arabinose (A), and l-Rhamnose (R). Points represent the average of 5 replicates, and the shaded bands indicate standard deviation.

To test whether increasing the availability of PC/pre-PC would improve PB production (Fig. 3), we introduced the plasmids constructed for heterologous expression of PC into the native producer *P. brasiliensis* Ab134. To this end, plasmids pVLDEF and pVLHIJK were conjugated into *P. brasiliensis* Ab134, both individually and in combination (Fig. 4A). All strains were cultured for three days in the presence of the required antibiotics and promoter inducers in the case of pVLDEF (pVLHIJK has a constitutive promoter). The cultures were extracted and analyzed by LC–MS. The AUC of the extracted ion chromatograms for the features of interest was compared across strains relative to the wild type (Fig. 4B).

Consistent with our hypothesis that the availability of PC may be limiting PB production, the presence of plasmid pVLDEF, containing the NRPS–PKS (*pppD*), 4′-phosphopantetheinyl transferase (*pppE*), and type II thioesterase (*pppF*) genes, resulted in a 3.45-fold improvement in the production of both PB analogues, while the amount of PA decreased (Fig. 4B). In contrast, the presence of plasmid pVLHIJK, which carries the accessory genes, led to a decrease in the production of all compounds. The combination of both plasmids resulted in an increase in the production of PB compared to the wild type, although not as pronounced as with pVLDEF alone (Fig. 4B). One possible explanation for decreased production is plasmid burden. Indeed, each plasmid individually caused a growth defect relative to the wild type when cultured in the presence of the antibiotics and inducers required for the production culture, and the combination of both plasmids resulted in an additive burden, leading to a substantially lower growth rate and total area under the growth curve in the first 24 h (Fig. 4C). An extended growth curve (72 h) showed that at ~40 hours, the absorbance of all the production cultures reached wild-type levels (Fig. S14).

We previously showed that vectors bearing green fluorescence protein are stably maintained in *P. brasiliensis* Ab134 even without selection for at least three days [25]. To discern the contribution of the antibiotics, plasmid maintenance, and gene expression to the growth defects observed, we generated growth curves under varied conditions (Fig. S15). The conclusion was that gene expression is the main contributor to the observed growth defect, given that (i) for pVLDEF, a statistically significant growth defect was observed only under induction conditions, but not after the addition of antibiotic or based on plasmid presence compared to the wild type (Fig. S15B), and (ii) for pVLHIJK (constitutive promoter), the same growth defect compared to the wild type was observed with or without kanamycin (Fig. S15C).

The combined production and growth results indicated that expression of *pppDEF* (but not *pppHIJK*) was limiting PB production. The growth defect conferred by gene expression from each plasmid is comparable (Fig. 4C), but only pVLDEF resulted in improved PB production (Fig. 4B).

### Pseudovibriamide B isolation

After establishing that *P. brasiliensis* Ab134 pVLDEF was the most proficient producer of PB, this strain was selected for scaled-up cultivation. A total of 12 liters of culture were extracted using XAD-7HP resin and methanol, yielding 16.7 g of crude extract. The crude extract was desalted using a C18 SPE cartridge (3.42 g) and then fractionated on a C18 cartridge using a Combiflash system (334 mg). Thirty-four milligrams of the Combiflash fraction were aliquoted for screening bioassays. Since this fraction contains all pseudovibriamides as a mixture, it will be referred to as Pmix from now on (Fig. S2).

The remaining 300 mg of the Combiflash fraction were further separated by size-exclusion chromatography on a Sephadex LH-20 gravity column. Out of the 30 methanol fractions collected, PB analogues were most abundant in fraction 9 (33.8 mg) (Figs. S3–S4). Finally, LH-20 fraction 9 was separated by semi-preparative C18 HPLC (Fig. S5). LC–MS/MS analysis of four collected fractions revealed that fraction B corresponded to PB1 (3 mg), and fraction D corresponded to a mixture of PB2 and PB3 (3.6 mg) (Figs. S6–S8). The overall workflow for compound isolation was composed of five steps (Fig. S9).

### MAC assay

**Figure 5:**
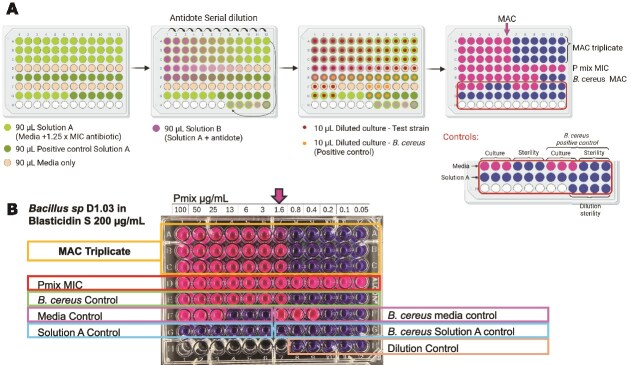
MAC assay. (A) MAC assay scheme. Solution A: media + antibiotic. Solution B: media + antibiotic + antidote. MAC plate design. Rows A-C: MAC triplicate. Row D: antidote control without antibiotics. Row E: protection positive control (*B. cereus* and blasticidin S). Row F: culture control and media sterility without antibiotics. Row G: culture control and media sterility with antibiotics. Row H: sterility control of the serial dilution. (B) MAC results for sponge isolate *Bacillus* sp. D1.03 in blasticidin S 200 μg/ml with serial dilution of Pmix starting at 100 μg/ml.

To measure antidote activity using marine sponge isolates, we developed an assay, termed MAC, which consists of a modified MIC assay in a 96-well plate format (Fig. 5A). The MAC assay consists of a serial dilution of the antidote across columns of the 96-well plate, with all wells containing a constant concentration of antibiotic. The concentration of antibiotic used was 1.25× the measured MIC for the antibiotic. However, MIC values can vary up to twofold among replicates [26], and 1.25× the initially observed MIC was not sufficient to prevent the growth of 5 of the 12 strains tested when performing the MAC. For those cases, the antibiotic concentration used for the MAC assay was individually optimized to ensure it was high enough to fully inhibit bacterial growth in the absence of the antidote (Table 1). Under these conditions, no bacterial growth is expected in any well unless the concentration of antidote is sufficient to protect the bacteria. After the incubation period, resazurin is added to each well to assess cell viability.

**Table 1:** Strains tested for the antidote activity of pseudovibriamides. Source of each strain, MIC against blasticidin S (each in triplicate), concentration of blasticidin S used for MAC assay, protection observed with Pmix in the MAC assay using up to 100 μg/ml Pmix, protection observed with pure PB1 and PB2/3 in MAC assay (range from 2 independent assays each in triplicate).

Strain	Strain information	Blasticidin S MIC (μg/ml)	Blasticidin S concentration for MAC (μg/ml)	Pmix MAC (μg/ml)	PB 1 MAC (μg/ml)	PB 2 MAC (μg/ml)
*Pseudovibrio brasiliensis* Ab134	Marine sponge isolate	512	640	no protection	-	-
***Bacillus cereus* NRRL B-3711**	Soil isolate	128–155^*^	175	1.6	0.31–0.63	0.63–1.25
*Pseudoalteromonas agarivorans* NW4327	Sponge pathogen	256	300	no protection	-	-
*Vibrio coralliilyticus* BAA-450	Coral pathogen	512	600	no protection	-	-
***Bacillus* sp. D1.03**	Marine sponge isolate	128–175^*^	200	1.6	0.63	0.63
*Bacillus* sp. D1.08	Marine sponge isolates	256–300^*^	350	no protection	-	-
*Bacillus* sp. D1.09		256	300	no protection	-	-
*Bacillus firmus* D2.07		256–350^*^	400	no protection	-	-
*Shewanella* sp. D2.04		375	450	no protection	-	-
*Sphingobium yanoikuyae* D2.09		512	600	no protection	-	-
*Staphylococcus* sp. D2.19		256–350^*^	400	no protection	-	-
*Staphylococcus haemolyticus* D2.20		375	450	no protection	-	-

^*^The higher values in the MIC range were observed in secondary assays with optimized, <2-fold dilutions of blasticidin S.

Before performing the MAC assay, we first needed to establish the MIC of blasticidin S against the bacterial strain of interest. We began with *B. cereus*. The observed MIC of blasticidin S against *B. cereus* in marine broth was 128 μg/ml. Note that this is one dilution below the MIC observed in spent media (Fig. 2B) as MIC is mostly a relative value and can vary depending on culture conditions and the starting culture used [26]. The minimum concentration of Pmix antidote capable of protecting *B. cereus* against 175 μg/ml of blasticidin S was 1.6 μg/ml (Table 1).

### Pseudovibriamides can protect a *Bacillus* sp. marine sponge isolate against blasticidin S

With the MAC assay established, we began screening the antidote activity using Pmix against blasticidin S with sponge isolates, in addition to *P. brasiliensis* Ab134 itself. To assess activity in strains relevant to the environmental contexts of *Pseudovibrio*, we included nine strains of marine sponge bacteria isolated from sponges from which *Pseudovibrio* containing the *ppp* BGC had also been isolated [27, 28]. The marine sponge isolates were authenticated by sequencing the 16S rRNA gene of each isolate (Table S6). The 16S sequences were also compared against the Sponge Microbiome Project 16S sequences database (SpongeEMP) [29], which showed that similar strains were observed in the metagenomic data collected from other marine sponges worldwide (Table S8). In addition, we included the sponge pathogen *Pseudoalteromonas agarivorans* NW4327 [30]. Given the paucity of characterized sponge pathogens, we also added the coral pathogen *Vibrio coralliilyticus* BAA-450 [31], since *Pseudovibrio* has also been isolated from corals [17].

The MIC of blasticidin S in marine broth was determined for each of the 12 strains (Table 1). Based on these MIC values, we performed the MAC assay using Pmix to screen antidote activity across all strains (Fig. 5B, Table 1). Neither of the two pathogens nor most sponge isolates were protected. Only one of the marine sponge isolates was protected, *Bacillus* sp. D1.03, with a MAC of 1.6 μg/ml of Pmix against 200 μg/ml of blasticidin S (Fig. 5B).

After identifying strains that could be protected by Pmix, the two responsive strains were further tested with purified PB1 and the mixture of PB2 and PB3. *B. cereus* was protected against 175 μg/ml of blasticidin S with as little as 0.31–0.63 μg/ml of PB1 and 0.63–1.25 μg/ml of PB2/3. The marine sponge isolate *Bacillus* sp. D1.03 was protected against 200 μg/ml of blasticidin S with 0.63 μg/ml of each PB analogue (Table 1). This low ratio of PB to blasticidin S is comparable to what has been reported for detoxin D1 [15, 32].

Additionally, the MAC of Pmix was tested with *B. cereus* NRRL B-3711 at 12 different concentrations of blasticidin S (from 175 to 450 μg/ml) to establish the dose response. The increase in Pmix required to rescue *B. cereus* in the presence of higher blasticidin S concentrations was non-linear, approaching an exponential dose response up to 350 μg/ml (Fig. S16). Antidote activity was not observed in concentrations higher than 350 μg/ml, coinciding with the high blasticidin S concentrations used for most strains that were not protected by pseudovibriamides.

To determine whether the antidote activity was reproducible in co-culture conditions, the protected strain, *Bacillus* sp. D1.03 was co-cultured with *P. brasiliensis* A134 wild type and with the ∆*pppE* mutant, which does not produce pseudovibriamides [18]. After 5 hours of co-cultivation, the cultures were treated with blasticidin S at 1.25× the MIC determined for *Bacillus* sp. D1.03*.* Colony-forming units (CFU) were then quantified and compared to untreated controls (Fig. S17). The CFU counts of *Bacillus* sp D1.03 were not significantly affected by blasticidin S when co-cultured with the wild-type producer of PB. However, the CFU of *Bacillus* sp D1.03 decreased 86% when co-cultured with the ∆*pppE* mutant in the presence of blasticidin S (Fig. S17).

## Discussion

**Figure 6:**
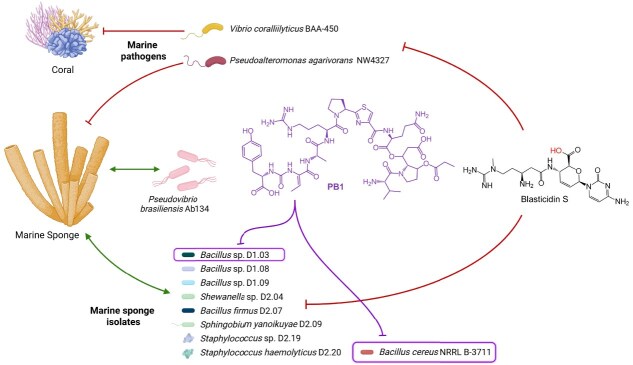
Summary of PB antibiotic antidote results. Pseudovibriamide B selectively protected the marine sponge isolate *Bacillus* sp. D1.03 and the soil isolate *B. cereus* NRRL B-3711 against blasticidin S. No protection was observed for a marine sponge and a coral pathogen, for *P. brasiliensis* itself and for eight marine sponge isolates tested, all of which showed higher MIC values for blasticidin S (Table 1).

In this study, we advanced the understanding of pseudovibriamides biosynthesis and their putative ecological role as antibiotic antidotes produced by *P. brasiliensis* Ab134 (Fig. 6). Based on previous studies that assigned the genes responsible for the biosynthesis of pseudovibriamides [16, 18], we first established a workflow to improve production and purification of these compounds. Deletion and overexpression of the predicted two-component system *pppOP* had only a modest impact on pseudovibriamide production (Fig. S10), suggesting that either this system does not regulate the BGC directly or that regulation is mediated through more complex or context-dependent pathways not recapitulated under our assay conditions.

Despite the unsuccessful attempts to heterologously express the *ppp* BGC, the constructed plasmids were used for improving PB production in the native host. Overexpressing the core biosynthetic genes *pppDEF* in *P. brasiliensis* Ab134 improved the production of PB analogues over threefold (Fig. 4). Additionally, this result supported the hypothesis that PC (or pre-PC) acts as a nucleophile to release PA from the TE domain in module 7 resulting in PB (Fig. 3) as recently demonstrated for the structurally related chitinimides [33]. The results also supported the hypothesis that the low availability of PC (or pre-PC) was limiting the production of PB. Interestingly, a close analogue of the linear peptide PA named pseudobulbiferamide was recently reported from *Microbulbifer* γ-Proteobacteria isolated from marine sponges. Despite containing the PKS-NRPS homologue to *pppD*, the full-length depsipeptide analogue of PB was not detected, indicating that PKS-NRPS availability may also be limiting depsipeptide production in this strain [34]. The methods for liquid cultivation, extraction, and isolation of PB were also optimized, leading to a reproducible and more simplified workflow (Fig. S9) compared to the previous cultivation in solid media and longer workflow [16].

Previous studies with detoxins, rimosamides, and chitinimides used a paper disk diffusion method for antidote analysis [13, 14, 32]. The developed 96-well plate MAC assay proved to be a reproducible and quantitative tool for evaluating antibiotic antidote activity (Fig. 5). Our results showed that PB has selective antidote activity, with protection observed for *B. cereus* and for a marine sponge isolate, *Bacillus* sp. D1.03. In contrast, two marine pathogenic strains and seven other sponge isolates were not protected by PB (Table 1), suggesting that the antidote activity is selective and of narrow spectrum. Thus, PB turned out to be narrower spectrum than we initially hypothesized, as it protected only one of the sponge isolates tested (Fig. 6). Of note, the blasticidin S MIC for protected bacteria was lower than for non-protected ones (including the two other *Bacillus* strains that were not protected), indicating that the non-protected strains already have more natural resistance against blasticidin S.

Together, these findings expand our understanding of pseudovibriamide biosynthesis and biological activity. The scalable production now provides a foundation for future mechanistic studies and for exploring the potential application of pseudovibriamides as selective antidote agents. Future work will focus on establishing the mechanism of antidote activity and assessing whether this activity can be translated into biotechnological or therapeutic applications.

## Supplementary Material

SI_fixed_refs_v2_ycag014

## Data Availability

All data generated or analyzed during this study are included in this published article and its supplementary information file.
