# Supplementary material for: Pseudovibriamide B from marine sponge-associated bacteria acts as a selective antibiotic antidote
Source: ISME Commun. 2026 Jan 22;6(1):ycag014. doi: 10.1093/ismeco/ycag014 (PMC12911931; doi:10.1093/ismeco/ycag014)
Supplement: SI_fixed_refs_v2_ycag014 [file si_fixed_refs_v2_ycag014.pdf]

Supplementary Information

# Pseudovibriamide B from marine sponge-associated bacteria acts as a selective antibiotic antidote

Vitor Lourenzon[1], Yitao Dai[1], Mandisa Timba[1], Subhash Yadav[2], Gav Mingolelli[1], Matthew Henke[1], Detmer Sipkema[2], Alessandra S. Eustaquio[1*].

[1]Department of Pharmaceutical Sciences and Center for Biomolecular Sciences, Retzky College of Pharmacy, University of Illinois Chicago, USA

[2]Laboratory of Microbiology, Wageningen University, the Netherlands

*Correspondence: ase@uic.edu

**Table of Contents**

SUPPLEMENTARY RESULTS ........................................................ 3

A two-component system could not be harnessed to increase pseudovibriamide production. ....... 3

SUPPLEMENTARY METHODS........................................................ 4

Plasmids construction. ........................................................ 4

Plasmid transfer into bacterial strains ........................................................ 5

In-frame deletion of *pppO*........................................................ 6

LC-MS analysis ........................................................ 6

Purification and structure confirmation of pseudovibriamide B. ........................................................ 7

TABLES ........................................................ 10

Table S1. Isolation source of bacterial strains used in this work. ........................................................ 10

Table S2. Culture conditions for testing pseudovibriamides production. ........................................................11

Table S3. PCR primer description. ........................................................ 12

Table S4. Plasmid description. ........................................................ 13

Table S5. Antibiotics for plasmid selection. ........................................................ 14

Table S6. Strain authentication........................................................ 14

Table S7. Library of knock-out mutants of *P. brasiliensis* Ab134. ........................................................ 15

Table S8. Detection of the 16S rRNA sequence of the marine sponge–associated bacterium in the Sponge Microbiome Project dataset. ........................................................ 15

FIGURES........................................................ 16

Figure S1. Spent media antidote assay........................................................ 16

Figure S2. LCMS data of the Combiflash fraction named Pmix. ........................................................ 16

Figure S3. LCMS data of the 30 fractions collected from the LH-20 size exclusion chromatography separation. ........................................................ 17

Figure S4. LCMS data of the LH-20 fraction 9. ........................................................ 17

Figure S5. Semi-preparative HPLC separation. ........ 18

Figure S6. LCMS data of the four HPLC C18 fractions. ........ 18

Figure S7. LCMS data of the HPLC fraction F9.B. ........ 19

Figure S8. LCMS data of the HPLC fraction F9.D. ........ 20

Figure S9. Workflow of the isolation of pseudovibriamides B1 (V) and B2/3 (I/L). ........ 21

Figure S10. Testing the involvement of a two-component system in pseudovibriamide biosynthesis. ........ 22

Figure S11. LCMS data of the crude extract of Ab134 WT. ........ 23

Figure S12. LCMS data of the crude extract of *Burkholderia* sp FERM PB-3421 ∆*fr9A* pVLDEF pVLHIJK.. ........ 23

Figure S13. LCMS data of the crude extract of *E. coli* BL21 pVLDEF pVLHIJK. ........ 24

Figure S14. Growth curves of the four strains depicted in Figure 4A (main text).. ........ 24

Figure S15. Growth curves of the four strains shown in Figure 4A (main text) under different conditions. ........ 25

Figure S16. Dose-response correlation of the antidote activity under increasing concentrations of blasticidin S against *Bacillus cereus* NRRL B-3711. ........ 26

Figure S17. Co-culture assay of the protected strain *Bacillus* sp. D1.03 and *P. brasiliensis* Ab134 (WT and ∆*pppE*). ........ 27

**REFERENCES ........ 28**

## Supplementary Results

**A two-component system could not be harnessed to increase pseudovibriamide production.** Aiming to improve production of pseudovibriamides, we started by investigating putative regulatory systems that could be involved in the regulation of pseudovibriamide production. Two genes located approximately 3 kb upstream of the *ppp* BGC are predicted to encode a two-component system (TCS). Two-component systems are common global regulators composed of a sensor histidine kinase (*pppO*) and a response regulator (*pppP*). The histidine kinase, a transmembrane protein, detects specific environmental signals and auto phosphorylates a conserved histidine residue. This phosphate group is then transferred to an aspartate residue on the response regulator, which often functions as a transcription factor to modulate the expression of target genes [1].

To investigate whether the TCS plays a regulatory role in pseudovibriamide production, we generated a scarless in-frame deletion of *pppO*, which encodes the histidine kinase. Additionally, we cloned the TCS (*pppO* and *pppP*) into the vector pSEVA237R_Pem7, generating plasmid pMTX01, which was conjugated into *P. brasiliensis* Ab134 (**Figure S10A**). The strains were cultivated and extracted, and the extracts were analyzed by LC-MS. The area under the curve (AUC) of the extracted ion chromatograms was used for relative quantification of PB production (**Figure S10B**). Deletion of *pppO* decreased average PB1 titers to 72% and PB2/3 to 60% of the wild-type levels (see **Figure 1** for PB structures), whereas overexpression of *pppPO* increased average PB1 titers by 28% and PB2/3 by 12% compared to the empty vector (**Figure S10B**). However, most of these differences were not statistically significant. The only statistically significant change was the decrease of PB2/3 in the TCS deletion strain. These results indicate that the TCS system is either not implicated in pseudovibriamide biosynthesis or that it may potentially activate pseudovibriamide biosynthesis indirectly given the modest differences between mutants and wild type. The relative increase observed with overexpression of *pppPO* (compared to the empty plasmid) was of no practical use due to the lack of statistical significance and because the presence of the expression plasmid decreased the titers compared to the wild type by 26% for PB1 and 42% for PB2/3 (empty plasmid by 52% for PB1 and 54% for PB2/3) (**Figure S10B**).

## Supplementary Methods.

### Plasmids construction.

pVL001. Plasmid pVL001 was constructed as the vector backbone to later harbor *pppDEF*. We included two convergent inducible promoters to mimic the native, convergent gene organization of *pppD* and *pppEF*. pVL001 was built using 4 DNA fragments amplified from 4 different plasmids. The RK2 replicon, the tetracycline resistance marker (*tetA*), and the origin of transfer (*oriT*) were acquired from the plasmid pSEVA521 [2] The L-Arabinose inducible promoter system (*araC/*$P_{BAD}$) was amplified from the plasmid pHNF008 [3] . The L-Rhamnose inducible promoter system (*rhaSR/*$P_{rha}$) came from the plasmid pJeM1 [4]. And finally, the reporter *lacZα* was amplified from pYD004. The primers utilized for the amplification of each fragment are listed on **Table S3**. The HiFi DNA Assembly Master Mix was used for building pVL001 from purified PCR products and according to the manufacturer's instructions.

pVLDEF. The HiFi DNA Assembly Master Mix was used for building pVLDEF from two purified PCR products. The backbone of pVL001 was amplified excluding only the *lacZα* reporter gene. Additionally, *pppDEF* was amplified from the genomic DNA of Ab134.

pVLHIJK. The genes *pppHIJK* were amplified from the genomic DNA of Ab134. The PCR product and vector pAM4891 were digested with the restriction enzymes NdeI and BamHI. T4 DNA Ligase was used to ligate insert and vector resulting in pVLHIJK.

pMTX01. The genes *pppOP* were amplified from the genomic DNA of Ab134. The PCR product and vector pSEVA237R_Pem7 were digested with the restriction enzymes NcoI and BamHI. T4 DNA Ligase was used to ligate insert and vector resulting in pMTX01.

pMTD01. Homology arms for *pppO* in-frame deletion were PCR amplified from the genomic DNA of Ab134. Vector pYD004 was PCR amplified without the *mCherry* reporter gene. The HiFi DNA Assembly Master Mix was used for building pMTD01 from purified PCR products.

**Plasmid transfer into bacterial strains**

Plasmid electroporation into *E. coli* strains. To make electrocompetent cells, a fresh culture of *E. coli* S17-1 was started by inoculating 1 mL of an overnight seed culture into 50 mL of LB in a 250 mL Erlenmeyer flask and incubating at 37 °C, 200 rpm, until an optical density at 600 nm ($OD_{600}$) of 0.4 to 0.6 was reached. Cells were harvested by centrifugation at 4 °C, 4000 rpm for 10 min. The cells were washed twice with ice-cold 10% glycerol solution, the first wash with 50 mL and the second with 25 mL. Finally, the *E. coli* cells were resuspended in 1 mL of the ice-cold 10% glycerol solution and aliquoted to 50 μL for storage at -80 °C until use. The constructed plasmids (~100 ng) were transformed into *E. coli* S17-1 (50 μL suspension) using electroporation (2.5kV, 200Ω, time constant 5 ms).

Plasmid conjugation into *Pseudovibrio brasiliensis* Ab134. All the plasmids introduced into Ab134 were first electroporated into *E. coli* S17-1, which was then used as the conjugation donor for introducing plasmids into Ab134. The selection for each plasmid was done using antibiotics as listed in **Table S5**. For conjugation, fresh cultures of Ab134 WT and *E. coli* S17-1 containing a plasmid were started by inoculating 400 μL of overnight seed culture into 20 mL of MB and 20 mL of LB (with appropriate antibiotic), respectively. The cultures were incubated at 30 °C, 200 rpm, until $OD_{600}$ of 0.4 to 0.6 was reached. Cells were harvested by centrifugation at 4 °C, 4000 rpm for 5 min. Ab134 cells were resuspended in 2 mL of MB. *E. coli* cells were washed twice with 10 mL LB to completely remove the antibiotics before the cells were resuspended in 2 mL of LB for conjugation. 500 μL each of Ab134 and *E. coli* S17-1 cell suspensions were mixed in a 1.5 mL tube and then plated into two MA plates. Negative control plates were inoculated with 250 μL of each cell suspension of Ab134 and *E. coli* S17-1 separately.

The plates were incubated at 30 °C for 18 to 20 hours. The cell mass was harvested from the conjugation plate using a 10 μL loop and streaked into a MA selection plate containing the appropriate antibiotic for plasmid selection (**Table S5**) and carbenicillin (50 μg/mL) to selectively kill *E. coli*. The plates were incubated at 30 °C for 18 to 20 hours. Single colonies growing on the selection plates were picked and cultured in 5 mL of MB containing antibiotic for the desired plasmid. Cultures were incubated at 30 °C, 200 rpm, for 18 – 20 hours. Plasmids were extracted from 2-ml aliquots and confirmed by restriction digestion with adequate restriction enzymes (**Table S4**).

Plasmid conjugation into *Burkholderia sp.* FERM BP-3421 Δ*fr9A*. All the plasmids introduced to FERM BP-3421 were first electroporated into *E. coli* S17-1, which was then used as the conjugation donor. The selection for each plasmid was done using appropriate antibiotics (**Table S5**). For conjugation, fresh cultures of FERM BP-3421 and *E. coli* S17-1 containing plasmids were started by inoculating 400 µL of overnight seed culture into 20 mL of LB (with appropriate antibiotic for the *E. coli* containing plasmids). The cultures were incubated at 30 °C, 200 rpm, until $OD_{600}$ of 0.4 to 0.6 was reached. Cells were harvested by centrifugation at room temperature, 4000 rpm for 5 min. The *E. coli* cell pellet was washed twice with 10 mL of LB. The FERM BP-3421 and *E. coli* cells were resuspended in 1 mL of LB. Conjugation was performed as described for Ab134. The selection for FERM BP-3421 with the desired vector were performed using the appropriate antibiotic for the plasmid, and gentamycin (10 µg/mL) to selectively kill *E. coli*. Plasmid extraction and confirmation was performed as described for Ab134.

**In-frame deletion of *pppO*.**

In-frame deletion was performed by homologous recombination as described by (Dai, Lourenzon, et al., 2024) using plasmid pMTD01 containing homology arms designed to delete 990 bp from the *pppO* gene (71% of the gene).

**LC-MS analysis**

Sample preparation for LC-MS analysis. The crude extracts and fractions were resuspended in LC-MS grade methanol and diluted to a final concentration of 0.2 mg/mL to 0.5 mg/mL. Diluted samples were centrifuged for 5 min at 15000 rpm, and filtered using 0.2 µm, 4mm PTFE syringe filters (Thermo Scientific).

UPLC-ESI-ORBITRAP analysis. Analysis was conducted in UPLC-ESI-Orbitrap system (Vanquish HPLC and Orbitrap Exploris 120, Thermo Fisher Scientific) with a Split Sampler FT (VF-A10-A), binary pump F (VF-P10-A), UV/Vis detector (VF-D40-A) Column oven (VH-C10-A), switching valve (Rheodyne MXT715-004), and mass spectrometer orbitrap exploris 120. Two microliters of each sample were injected into a Poroshell 120 EC-C18 1.9 µm, 2.1 x 50 mm column (Agilent) kept at 30°C. Separation was carried out using a binary solvent system consisting of water with 0.1% formic acid (solvent A) and acetonitrile with 0.1% formic acid (solvent B), at a flow rate of 0.25 mL/min. The gradient program was as follows: 3% B at

0 min, increased to 5% B at 1 min, then to 40% B at 6 min, followed by a ramp to 95% B at 8 min. The system was held at 100% B for 1 min, then returned to 3% B at 11 min for re-equilibration.

UPLC-ESI-qTOF. The analysis of PC production by the heterologous expression hosts was performed on a Bruker Elute LC system coupled to a Bruker Compact qTOF mass spectrometer with electrospray ionization (ESI) as described in Dai 2024 [5] .

Relative quantification. Five µL of the XAD 7-HP resin extracts were aliquoted and solubilized in 50 µL of water. The solution was loaded into 100 mg C18 cartridges for solid phase extraction (SPE). One mL of water was then used to wash the column. One mL of water : methanol (1:1), and 2 mL of pure methanol was used for elution. The water : methanol and methanol fractions were gathered in pre-weighted vials and the solvent was removed under reduced pressure. The SPE fractions were then solubilized with LC-MS grade methanol to 0.5 mg/mL and analyzed in the UPLC-ESI-ORBITRAP as described above.

**Purification and structure confirmation of pseudovibriamide B.**

The crude extract (16.7 g) from 12 L of *P. brasiliensis* pVLDEF culture was solubilized in water at 1g/mL. 0.5 mL of the solution was injected into a Discovery DSC-18 SPE cartridge (5 g, 20 mL volume) at a time. The column was washed with 20 mL of water to remove excessive salt from MB. The sample was then eluted with 20 mL of 50% aqueous methanol and 40 mL of 100% methanol. PA and PB were present in both fractions (50% MeOH and 100% MeOH), so both fractions were gathered, and the solvent was removed under reduced pressure, leading to 3.42 g of the SPE fraction. The SPE fraction was then solubilized in 0.5 mL of methanol and added to 1 g of celite (Fisher C212) for injection in a 40 g HP C18 (RediSepRf) cartridge for dry injection. Flash chromatography was performed using a Teledyne Isco Combiflash system with a Hewlett-Packard Agilent 1100 system controller and pumps with a Model G1315A diode array detector (DAD) and Shimadzu ELSD LTII light scattering detector. The run consisted of a 20-minute gradient from 20 to 100% methanol with a 40 mL/min flow. Pseudovibriamides eluted between 40-45% methanol as indicated by LC-MSMS analysis, resulting in 334 mg of the Combiflash fraction. This fraction was active as an antibiotic antidote, protecting *B. cereus* NRRL B-3711 against 175 µg/mL of

blasticidin S in concentrations as low as 1.6 µg/mL. Therefore, 34 mg of the Combiflash fraction (named Pmix) was aliquoted for antidote activity screening assays.

The remaining 300 mg of the Pmix fraction was loaded into a gravity column (120 cm tall and 4.6 cm internal diameter) with 400 g of Sephadex LH-20 (Cytiva) packed with methanol for a size exclusion chromatography. Elution was performed with methanol at 1.3 mL/min flow for 26 hours. Fraction collection started 3 hours after the start, and 180 9 mL tubes were collected. The tubes were gathered 6 by 6, leading to 30 fractions (**Figure S3**). According to LC-MSMS analysis, fraction 9 (33.8 mg) contained a mixture of PB1, PB2/3, and a few additional peaks (**Figure S4**). This fraction was subjected to a final round of separation using reverse phase HPLC Agilent 1260 infinity system with a 1260 Quaternary Pump (G1311B), Autosampler 1260 ALS (G1329B), and UV/Vis detector 1260 DAD VL (G1315C). After solubilization in aqueous 10% HPLC grade methanol with a final concentration of 10 mg/mL. In total, 35 injections of 80 µL was performed in a semi-preparative Kinetex 5 µm C18 (250 × 10 mm) (Phenomenex). Chromatographic separation was performed using a binary solvent system consisting of water with 0.1% formic acid (solvent A) and acetonitrile with 0.1% formic acid (solvent B), at a flow rate of 4.6 mL/min. The gradient elution was programmed as follows: 3% B at 0 min, increased to 5% B over 3 min, then to 15% B at 6 min, followed by an isocratic hold at 15% B for 12 min. The gradient was then ramped to 100% B over 2 min, held at 100% B for 2 min, and returned to 3% B over 2 min. A re-equilibration period of 5 min at 3% B was applied before the next injection.

The four major peaks were collected (**Figure S5**) and analyzed by LC-MSMS (**Figure S6**). Based on accurate mass and the fragmentation pattern, we confirmed that fraction F9-B is pseudovibriamide B1 (3 mg) and F9-D is a mixture of pseudovibriamide B2 and B3 (3.4 mg) (**Figure S7 – S8**).

**Antidote activity in co-culture conditions.**

The protected strain *Bacillus* sp. D1.03 was cultured along with *P. brasiliensis* Ab134 WT or the pseudovibriamide-defective Δ*pppE* mutant (**Figure S17**). Overnight seed cultures of each strain were used to inoculate fresh 5 mL MB, which was then incubated at 30 °C until an $OD_{600}$ of 0.1 was reached. Once all three strains reached the targeted $OD_{600}$, four co-culture tubes were prepared with 5 mL of fresh MB

each. *Bacillus* sp. D1.03 (100 µL) was added to all four tubes, and *P. brasiliensis* Ab134 (25 µL) was added as either the WT (two tubes) or the Δ*pppE* mutant (two tubes). The co-cultures were incubated at 30 °C and 200 rpm for 5 hours, after which blasticidin S was added to two tubes (one WT and one Δ*pppE*) to a final concentration of 160 µg/mL (1.25 × the MIC determined for *Bacillus* sp. D1.03). After adding the antibiotic, incubation was resumed for 18 h. The co-cultures were serially diluted to $10^{-6}$, and 50 µL aliquots were plated on MA (20 mL). The plates were incubated at 30 °C for 2 days, allowing *P. brasiliensis* Ab134 colonies to develop a brown pigmentation, enabling distinction between the two species and CFU/mL determination.

## Tables

**Table S1. Isolation source of bacterial strains used in this work.**

| Isolate | Source | Reference |
|---|---|---|
| *Pseudovibrio brasiliensis* Ab134 | Marine sponge *Arenosclera brasiliensis* | Rua CPJ et al. 2014 [6] |
| *Bacillus cereus* NRL B-3711 (ATCC 14579) | Multiple sources * (commun in the soil) | Frankland and Frankland 1887 [7] |
| *Pseudoalteromonas agarivorans* NW4327 | Sponge pathogen | Webster NS et al 2002 [8] |
| *Vibrio coralliilyticus* BAA-450 | Coral pathogen | Ben-Haim Y et al. 2003 [9] |
| *Bacillus* sp. D1.03 | Marine sponge *Aplysina aerophoba* | Versluis et al. 2017 [10] |
| *Bacillus* sp. D1.08 | Marine sponge *Petrosia ficiformis* | Versluis et al. 2017 [10] |
| *Bacillus* sp. D1.09 | Marine sponge *Aplysina aerophoba* | Versluis et al. 2017 [10] |
| *Bacillus firmus* D2.07 | Marine sponge *Xestospongia muta* | Indraningrat et al. 2019 [11] |
| *Shewanella* sp. D2.04 | Marine sponge *Xestospongia muta* | Indraningrat et al. 2019 [11] |
| *Sphingobium yanoikuyae* D2.09 | Marine sponge *Xestospongia muta* | Indraningrat et al. 2019 [11] |
| *Staphylococcus* sp. D2.19 | Marine sponge *Agelas sventres* | Indraningrat et al. 2019 [11] |
| *Staphylococcus haemolyticus* D2.20 | Marine sponge *Agelas sventres* | Indraningrat et al. 2019[11] |

**Bacillus cereus* NRRL-B-3711 was originally isolated from the air in a cow shed [7]. *B. cereus* is also frequently isolated from soil, food, and marine sponges [12, 13].

**Table S2. Culture conditions for testing pseudovibriamides production.** Media and supplementation used in the engineered native producer *P. brasiliensis* Ab134 and in the heterologous hosts *E. coli* BL21 and *Burkholderia* sp. FERM BP-3421 ∆*fr9A*.

| Strain | Media | Suplementation |
|---|---|---|
| *P. brasiliensis* Ab134 | MB | - |
| *P. brasiliensis* Ab134 pVLDEF | MB | Tet (100 µg/mL) + L-Ara (100 mM) + L-Rha (50 mM) |
| *P. brasiliensis* Ab134 pVLHIJK | MB | Kan (200 µg/mL) |
| *P. brasiliensis* Ab134 pVLDEF pVLHIJK | MB | Tet (100 µg/mL) +Kan (200 µg/mL) + L-Ara (100 mM) + L-Rha (50 mM) |
| *E. coli* BL21 pVLDEF pVLHIJK | LB | Tet (10 µg/mL) +Kan (50 µg/mL) + L-Ara (100 mM) + L-Rha (50 mM) |
| *E. coli* BL21 pVLDEF pVLHIJK | Terific Broth | Tet (10 µg/mL) +Kan (50 µg/mL) + L-Ara (100 mM) + L-Rha (50 mM) |
| *Burkholderia* sp. FERM BP-3421 ∆*fr9A* pVLDEF pVLHIJK | LB | Tet (25 µg/mL) +Kan (500 µg/mL) + L-Ara (100 mM) + L-Rha (50 mM) |
| *Burkholderia* sp. FERM BP-3421 ∆*fr9A* pVLDEF pVLHIJK | 2S4G | Tet (25 µg/mL) +Kan (500 µg/mL) + L-Ara (100 mM) + L-Rha (50 mM) |
| *P. brasiliensis* Ab134 pMTX01 | MB | - |
| *P. brasiliensis* Ab134 Δ*pppO* | MB | - |

Tet, tetracycline. Kan, kanamycin. L-Ara, L-arabinose. L-Rha, L-rhamnose.

The sources of antibiotics and inducers were: tetracycline (Sigma-Aldrich), kanamycin (Biosynth Carbosynth), L-(+)-arabinose (Chem-Impex) and L-(+)-rhamnose (Ambeed).

**Table S3. PCR primer description**. Sequence and description of primers utilized for PCR. The predicted melting temperature (Tm) and the annealing temperature used for each set of primers is listed.

| Primer | Sequence 5' to 3' | Additional information | Tm ºC (Q5) | Annealing Temp ºC |
|---|---|---|---|---|
| **pVL001 backbone construction (Gibson assembly)** | | | | |
| SEVA521_Gib_F | GCATGATGAATCTTGGACTCCTGTTGATAG | Homology arm | 60 | 61 |
| SEVA521_Gib_R | TCGATGCAGGTGTGTGAAATTGTTATCCGC | Homology arm | 60 | |
| Pbad_Gib_F | ATTTCACACACCTGCATCGATTTATTATGAC | Homology arm | 58 | 59 |
| Pbad_Gib_R | CCATGGCATGAAACAGTAGAGAGTTGCG | Homology arm | 59 | |
| LacZ_Gib_F | TCTACTGTTTCATGCCATGGCGAGCTCTCAGCTTCGATGTAGGAG | Homology arm, NcoI, and SacI | 60 | 61 |
| LacZ_Gib_R | AATGAACAATCGGGGTACCCCGCTCGAGAGTTAGCTCACTCATTAGGC | Homology arm, KpnI, and XhoI | 61 | |
| Prha_Gib_F | GGGTACCCCGATTGTTCATTACGACCAGTC | Homology arm | 59 | 60 |
| Prha_Gib_R | GAGTCCAAGATTCATCATGCCGTTTGTG | Homology arm | 60 | |
| **pVLDEF construction (Gibson assembly)** | | | | |
| DEF.2_F | TCTACTGTTTAAGAACTCCCACACTCTTTC | Homology arm | 62 | 63 |
| DEF.2_R | AATGAACAATACACTCTCGACCTCCTATC | Homology arm | 62 | |
| VL001_F | TCGAGAGTGTATTGTTCATTACGACCAGTC | Homology arm | 59 | 60 |
| VL001_R | GGGAGTTCTTAAACAGTAGAGAGTTGCG | Homology arm | 59 | |
| **pVLHUJK construction (restriction ligation)** | | | | |
| pppK_F | GGAATTCCATATGTCAACCTAGAACTCCCACC | NdeI | 63 | 62 |
| pppH_comp_R | CGGGGATCCGAGGAGGTCGCATGATATC | BamHI | 62 | |
| **pMTX01 construction (restriction ligation)** | | | | |
| XpppOP_F | CGCGGATCCGCGAAACCTGTCACCTCCTTG | BamHI | 62 | 62 |
| XpppOP_R | CATGCCATGGCATGTGCTGCTCAATCTCATAGG | NcoI | 61 | |
| **pMTD01 construction (Gibson assembly)** | | | | |
| gib_YD004_F | ACACAACATACGAGCCGG | | 65 | 65 |
| gib_YD004_R | ACCCATCACATATACCTGCC | | 64 | |
| gib_UP_F | GGCAGGTATATGTGATGGGTACAAACCTGTCACCTCCTTG | Homology arm | 65 | 65 |
| gib_UP_R | GCTTCTCGATTGGAATATCGACGGTGACC | Homology arm | 64 | |
| gib_DOWN_F | CGATATTCCAATCGAGAAGCTCACAGAACG | Homology arm | 64 | 65 |
| gib_DOWN_R | TTCCGGCTCGTATGTTGTGTTGGTGAAATCTTCCAGCTCC | Homology arm | 65 | |
| **16S amplification for strain authentication** | | | | |
| 16S_27_F | AGAGTTTGATCMTGGCTCAG | | 62 | 63 |
| 16S_1492_R | CGGTTACCTTGTTACGACTT | | 62 | |

**Table S4. Plasmid description.** Plasmid composition, application in this work, and the restriction enzymes used to confirm each plasmid.

| Plasmid | Features | Description of features | Application | Restriction confirmation enzyme |
|---|---|---|---|---|
| pHNF008 | ori pBBR1 | Replicon | araC/PBAD used for pVL001 construction | - |
| | *araC/P$_{BAD}$* | P$_{BAD}$ - L-Arabinose inducible promoter | | |
| | Kan$^{R}$ | Marker - Kanamycin resistance gene | | |
| pJeM1 | ori pBBR1 | Replicon | rhaSR/Prha used for pVL001 construction | - |
| | Kan$^{R}$ | Marker - Kanamycin resistance gene | | |
| | *rhaSR/Prha* | rhaP$_{BAD}$ - L-Rhamnose inducible promoter | | |
| | *gfp* | Reporter (Green fluorescence protein) | | |
| pSEVA521 | *ori* RK2 | Replicon (compatible with *P. brasiliensis* Ab134 and the heterologous hosts). | ori RK2, tetA, and oriT used for pVL001 construction | - |
| | *tetA* | Marker - Tetracycline resistance gene | | |
| | *oriT* | Origin of conjugative transfer | | |
| pYD004 | *ori* R6K | Replication only in *E. coli* λpir | Used as a backbone for the construction of pMTD01 and as a source of lacZα for pVL001 construction | BamHI-HF- |
| | RP4 | Conjugation system | | |
| | CmR | Chloramphenicol resistance | | |
| | *sacB* | Counter selection marker utilizing sucrose | | |
| | *lacZα* | Reporter (blue-white screening) | | |
| pVL001 | *ori* RK2 | Replicon (compatible with *P. brasiliensis* Ab134 and the heterologous hosts) | Backbone with two inducible promoters for the construction of pVLDEF | EcoRV-HF |
| | *tetA* | Marker - Tetracyclin resistance gene. | | |
| | *oriT* | Origin of transfer | | |
| | *araC* | P$_{BAD}$ - L-Arabinose inducible promoter | | |
| | *rhaS*, and *rhaR* | rhaP$_{BAD}$ - L-Rhamnose inducible promoter | | |
| | *lacZα* | Reporter (blue-white screening). | | |
| pVLDEF (Based on pVL001) | *ori* RK2 | Replicon | Core biosynthetic genes for the heterologous / autologous expression of PC | EcoRV-HF |
| | *tetA* | Marker - Tetracyclin resistance gene | | |
| | *araC/P$_{BAD}$* | P$_{BAD}$ - L-Arabinose inducible promoter | | |
| | *rhaSR/Prha* | rhaP$_{BAD}$ - L-Rhamnose inducible promoter | | |
| | *pppDEF* | *lacZα* replaced with *pppDEF* (native RBS included) | | |
| pAM4891 | *ori* RSF1010 | Replicon (Compatible with *P. brasiliensis* Ab134 and the heterologous hosts) | Backbone for the construction of pVLHIJK | SamI |
| | Kan$^{R}$ | Marker - Kanamycin resistance gene | | |
| | Ptac | Constitutive promoter | | |
| | *gfp* | Reporter (Green fluorescence protein) | | |
| pVLHIJK (Based on pAM4891) | *ori* RSF1010 | Replicon (Compatible with *P. brasiliensis* Ab134 and the heterologous hosts). | Accessory genes for the heterologous / autologous expression of PC | SamI |
| | Kan$^{R}$ | Marker - Kanamycin resistance gene | | |
| | Ptac | Constitutive promoter | | |
| | *pppHIJK* | *gfp* replaced with *pppHIJK* (native RBS included) | | |
| pSEVA237R_Pem7 | *ori* pBBR1 | Replicon | Backbone for the construction of pMTX01 | |
| | Kan$^{R}$ | Marker - Kanamycin resistance gene | | |
| | PEM7 | Constitutive promoter | | |
| | *mCherry* | Reporter (Red fluorescence protein) | | |
| pMTX01 | *ori* pBBR1 | Replicon | Overexpression of the two component system | EcoRI-HF and NcoI-HF |
| | *kanR* | Marker - Kanamycin resistance gene. | | |
| | PEM7 | Constitutive promoter | | |
| | *pppOP* | mCherry replaced with *pppOP* (native RBS included) | | |
| pMTD01 | *ori* R6K | Replication only in *E. coli* λpir | Delivery of replacement allele for in-frame deletion of *pppO* | BamHI-HF |
| | RP4 | Conjugation system | | |
| | CmR | Chloramphenicol resistance | | |
| | *sacB* | Counter selection marker utilizing sucrose | | |
| | homology arms | lacZα replaced with upstream and downstream homology arms | | |

**Table S5. Antibiotics for plasmid selection.** List of antibiotics used to select strains containing the desired plasmids along with inducers used for pVLDEF.

| Plasmid | Promotor inducers | Strain | Antibiotic for Selection |
|---|---|---|---|
| pVLDEF | L-Arabinose 100 mM and L-Rhamnose 50 mM | *P. brasiliensis* Ab134 | Tetracycline 100 µg/mL |
| | | *E. coli* BL21 | Tetracycline 10 µg/mL |
| | | *Burkholderia* sp. FERM BP-3421 | Tetracycline 25 µg/mL |
| pVLHIJK | - | *P. brasiliensis* Ab134 | Kanamycin 200 µg/mL |
| | | *E. coli* BL21 | Kanamycin 50 µg/mL |
| | | *Burkholderia* sp. FERM BP-3421 | Kanamycin 500 µg/mL |
| pMTX01 | - | *P. brasiliensis* Ab134 | Kanamycin 200 µg/mL |

**Table S6. Strain authentication**. Authentication of the marine sponge isolates provided by the Sipkema's lab. Authentication was performed by BLAST using the 16S rRNA gene sequence amplified from each strain.

| Strain # | Code | Strain | 16 S Blast |
|---|---|---|---|
| DN51_2A1 | D1.03 | *Bacillus idriensis* | *Metabacillus sp/Bacillus sp.* |
| DN14_7A9 | D1.08 | *Bacillus stratosphericus* | *Bacillus altitudinis* |
| DN51_2A1 | D1.09 | *Bacillus indicus* | *Metabacillus sp/Bacillus sp.* |
| OLIGO-4.XM3 | D2.04 | *Shewanella sp.* | *Shewanella sp.* |
| Mucin-17.Xm4 | D2.07 | *Bacillus firmus* | *Bacillus firmus* |
| MA1/10-2.XM7 | D2.09 | *Sphingobium yanoikuyae* | *Sphingobium yanoikuyae* |
| GP-8.AS3 | D2.19 | *Staphylococcus epidermidis* | *Staphylococcus saprophyticus* |
| GP-6.AS3 | D2.20 | *Staphylococcus haemolyticus* | *Staphylococcus haemolyticus* |

**Table S7. Library of knock-out mutants of *P. brasiliensis* Ab134.** Production of pseudovibriamide A, B, and C (PA, PB, PC) by each mutant, and the assigned function of the deleted gene in each strain. YES* denotes analogues of the parent PB and PC, according to the gene function that was disrupted [5]

| Strain | PA | PB | PC | Gene Function |
|---|---|---|---|---|
| *P. brasiliensis* Ab134 WT | YES | YES | YES | - |
| *P. brasiliensis* Ab134 Δ*pppA* | - | - | YES | NRPS |
| *P. brasiliensis* Ab134 Δ*pppD* | YES | - | - | NRPS-PKS hybrid |
| *P. brasiliensis* Ab134 Δ*pppE* | - | - | - | 4'-Phosphopantetheinyl Transferase (4'-PPTase) |
| *P. brasiliensis* Ab134 Δ*pppF* | YES | YES | YES | Type-II Thioesterase (TE) |
| *P. brasiliensis* Ab134 Δ*pppG* | YES | YES | YES | Major Facilitator Superfamily (MFS) Transporter |
| *P. brasiliensis* Ab134 Δ*pppH* | YES | - | - | Acyltransferase - predicted to participate in the propionylation of the hydroxyroline incorporated on module 9 |
| *P. brasiliensis* Ab134 Δ*pppI* | YES | - | - | |
| *P. brasiliensis* Ab134 Δ*pppJ* | YES | - | - | |
| *P. brasiliensis* Ab134 Δ*pppK* | YES | YES* | YES* | Fe(II)-oxoglutarate dependent dioxygenase (hydroxylase) |

**Table S8. Detection of the 16S rRNA sequence of the marine sponge–associated bacterium in the Sponge Microbiome Project dataset.** The table summarizes the number of samples (out of 3,490) containing similar 16S sequences (≥97% identity threshold) and reports the host species and countries where the bacterium shows statistically significant enrichment (Fisher's exact test, $p < 0.05$) compared to all samples associated with the same host or country

| Bacteria strain | Present in x samples (x / 3490) | Significant enrichment in host | Significant enrichment in country |
|---|---|---|---|
| Pseudovibrio brasiliensis Ab134 | 189 | 8 | 7 |
| *Bacillus* sp. D1.03 | 19 | 0 | 0 |
| *Bacillus altitudinis* D1.08 | 15 | 0 | 0 |
| *Bacillus* sp. D1.09 | 19 | 0 | 0 |
| *Shewanella* sp. D2.04 | 4 | 0 | 0 |
| *Bacillus firmus* D2.07 | 15 | 1 | 2 |
| *Sphingobium yanoikuyae* D2.09 | 52 | 1 | 3 |
| *Staphylococcus saprophyticus* D2.19 | 87 | 6 | 2 |
| *Staphylococcus haemolyticus* D2.20 | 492 | 17 | 7 |

# Figures

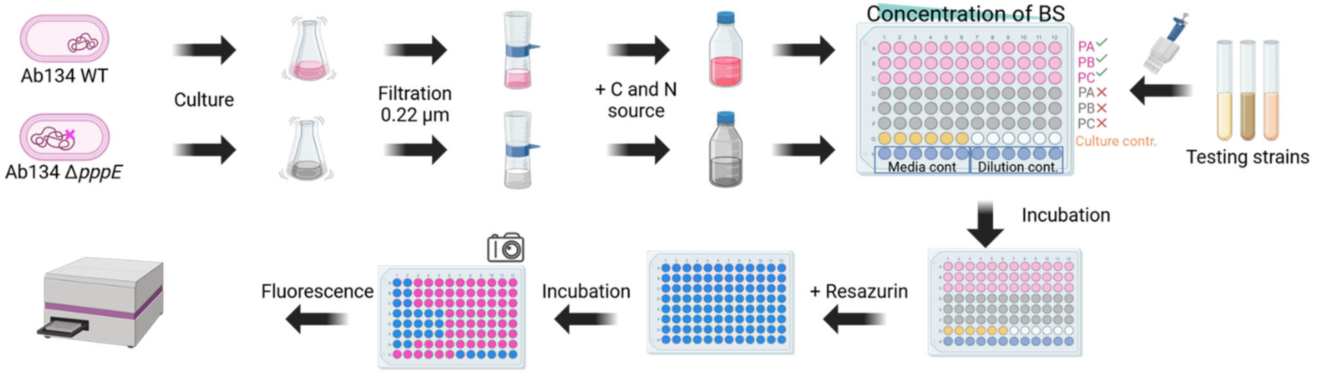


**Figure S1. Spent media antidote assay.** Workflow of the antibiotic antidote screening assay utilizing enriched, spent media from *P. brasiliensis* Ab134 wild type (WT) or the pseudovibriamide-deficient Δ*pppE* mutant.

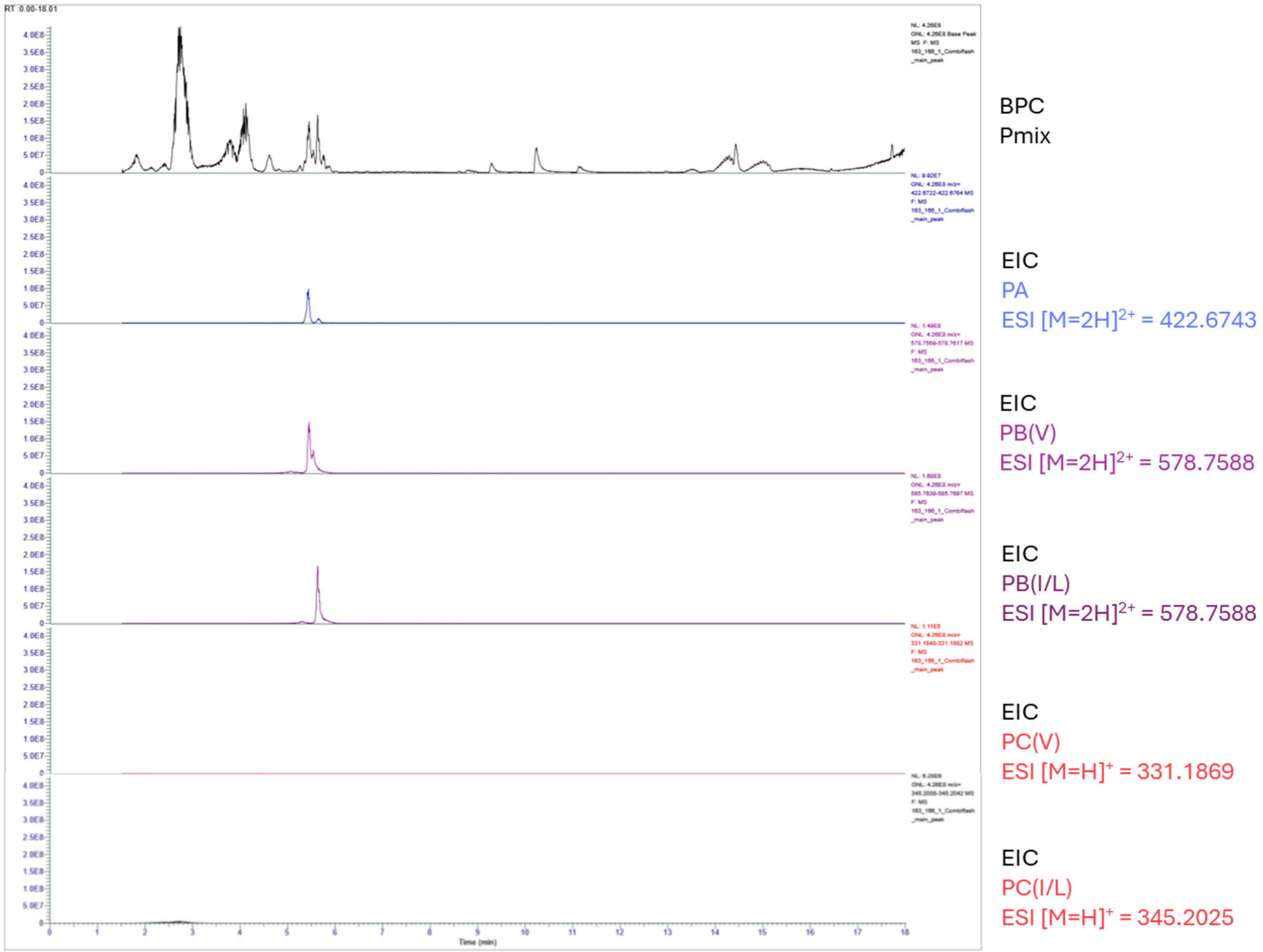


**Figure S2. LCMS data of the Combiflash fraction named Pmix**. The extracted ion chromatograms (EIC) show the presence of PA and PB1 (V) and PB2/3 (I/L) in Pmix.

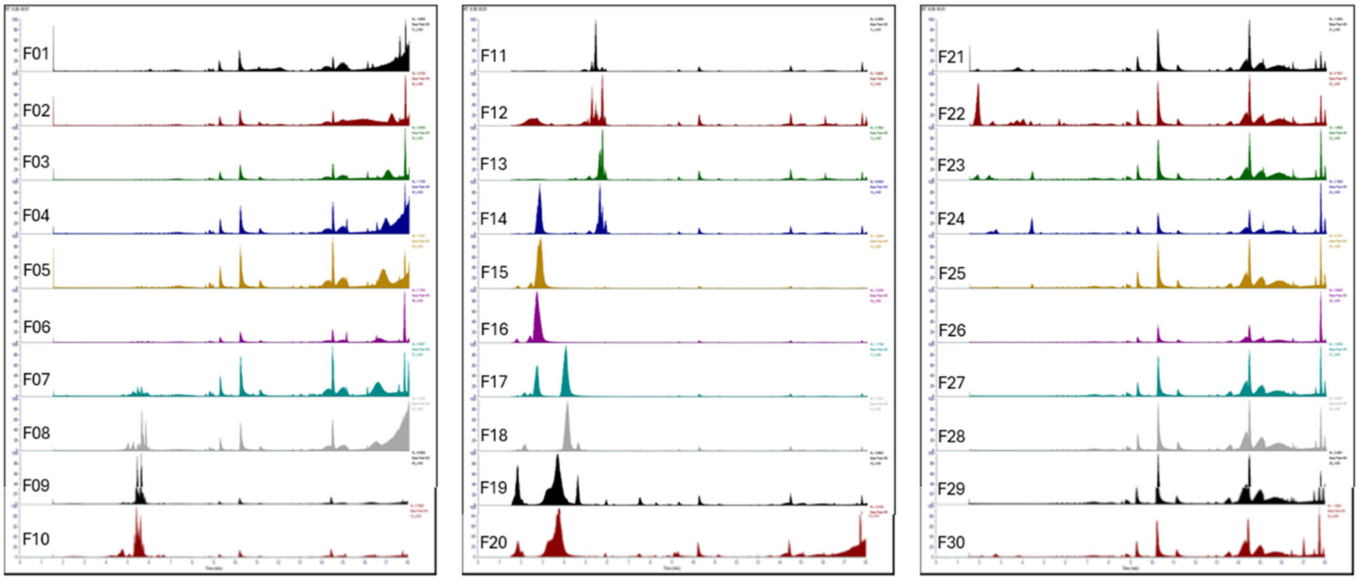


**Figure S3. LCMS data of the 30 fractions collected from the LH-20 size exclusion chromatography separation.** F1-F30, fractions 1-30 in methanol.

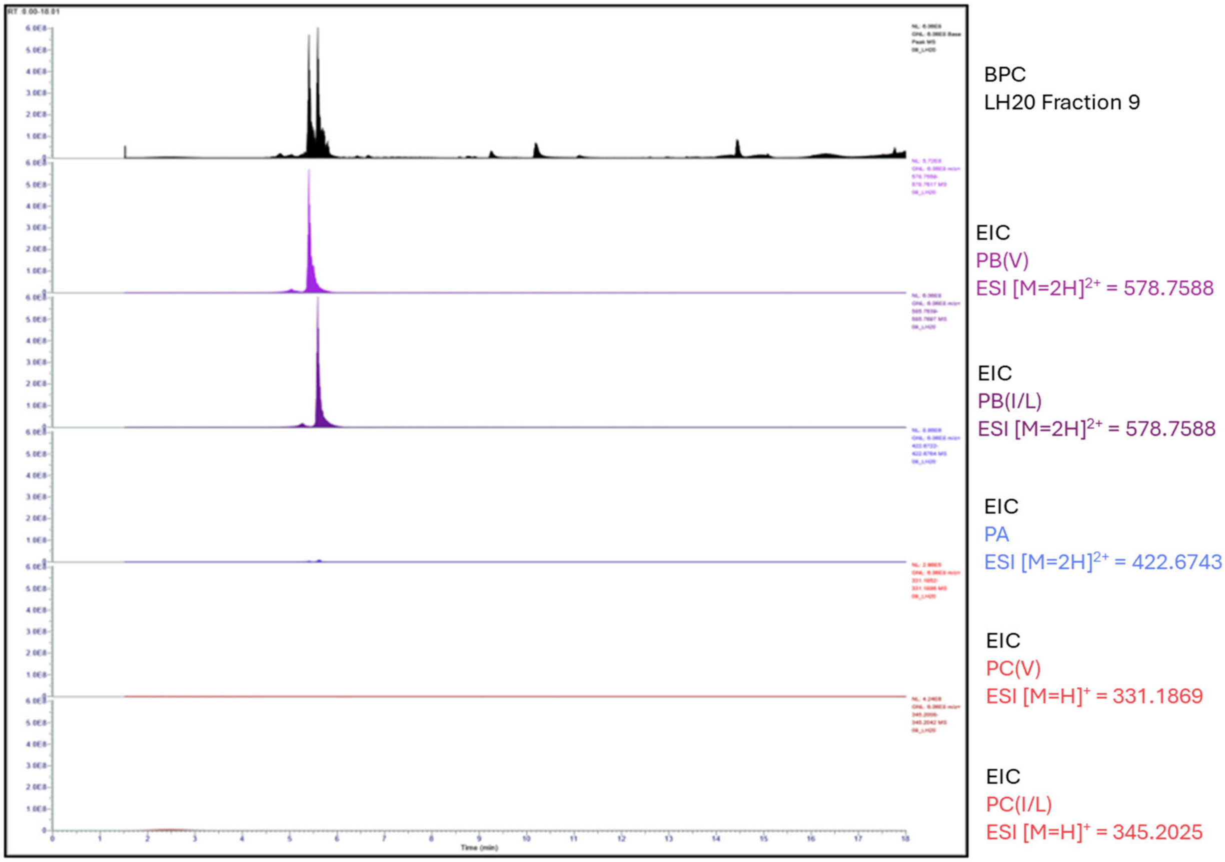


**Figure S4. LCMS data of the LH-20 fraction 9.** The extracted ion chromatograms (EIC) show the presence of PB1 (V) and PB2/3 (I/L). A minor trace of PA is also observed.

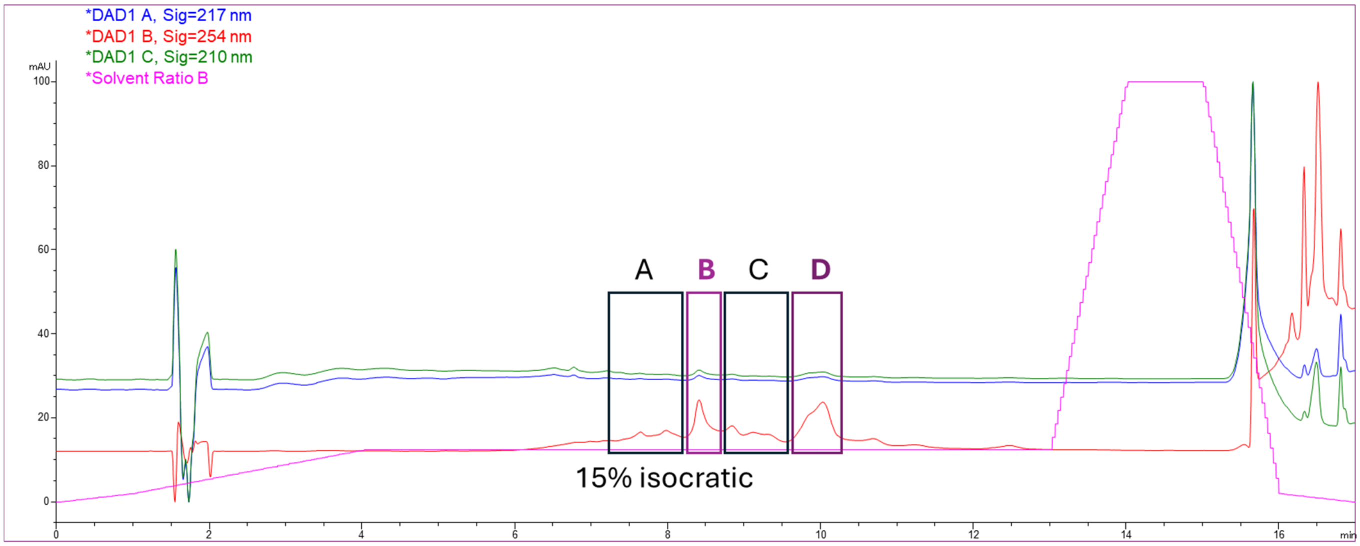


**Figure S5. Semi-preparative HPLC separation.** (**A**) unknown. (**B**) PB1 (V) (**C**) unknown. (**D**) PB2/3 (I/L). Compounds eluted in 15% aqueous acetonitrile with 0.1% formic acid.

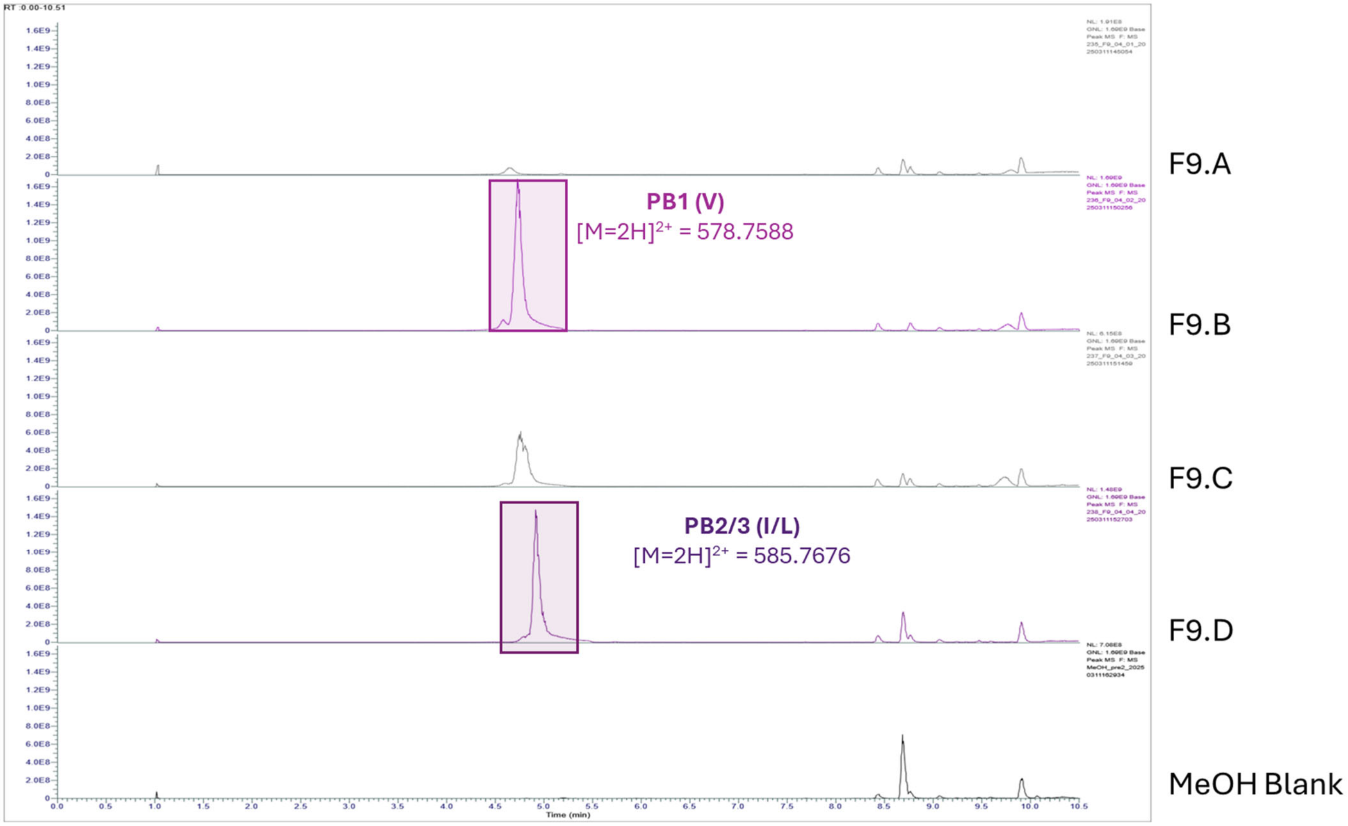


**Figure S6. LCMS data of the four HPLC C18 fractions.** F9.B corresponds to PB1 (V) and F9.D to PB2/3 (I/L).

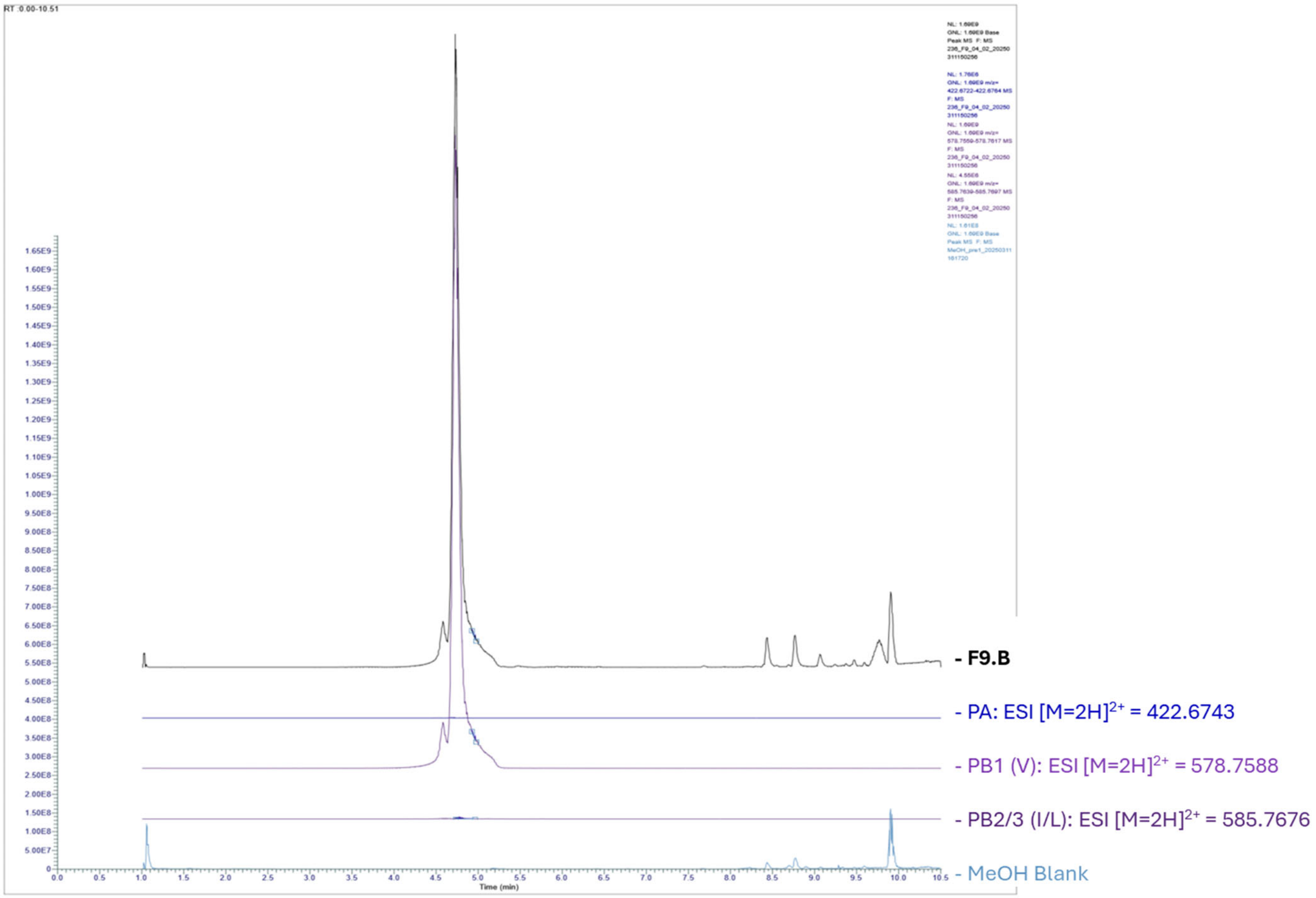


**Figure S7. LCMS data of the HPLC fraction F9.B.** The extracted ion chromatograms (EIC) confirmed the purity of PB1 (V) and absence of PA and PB2/3 (I/L).

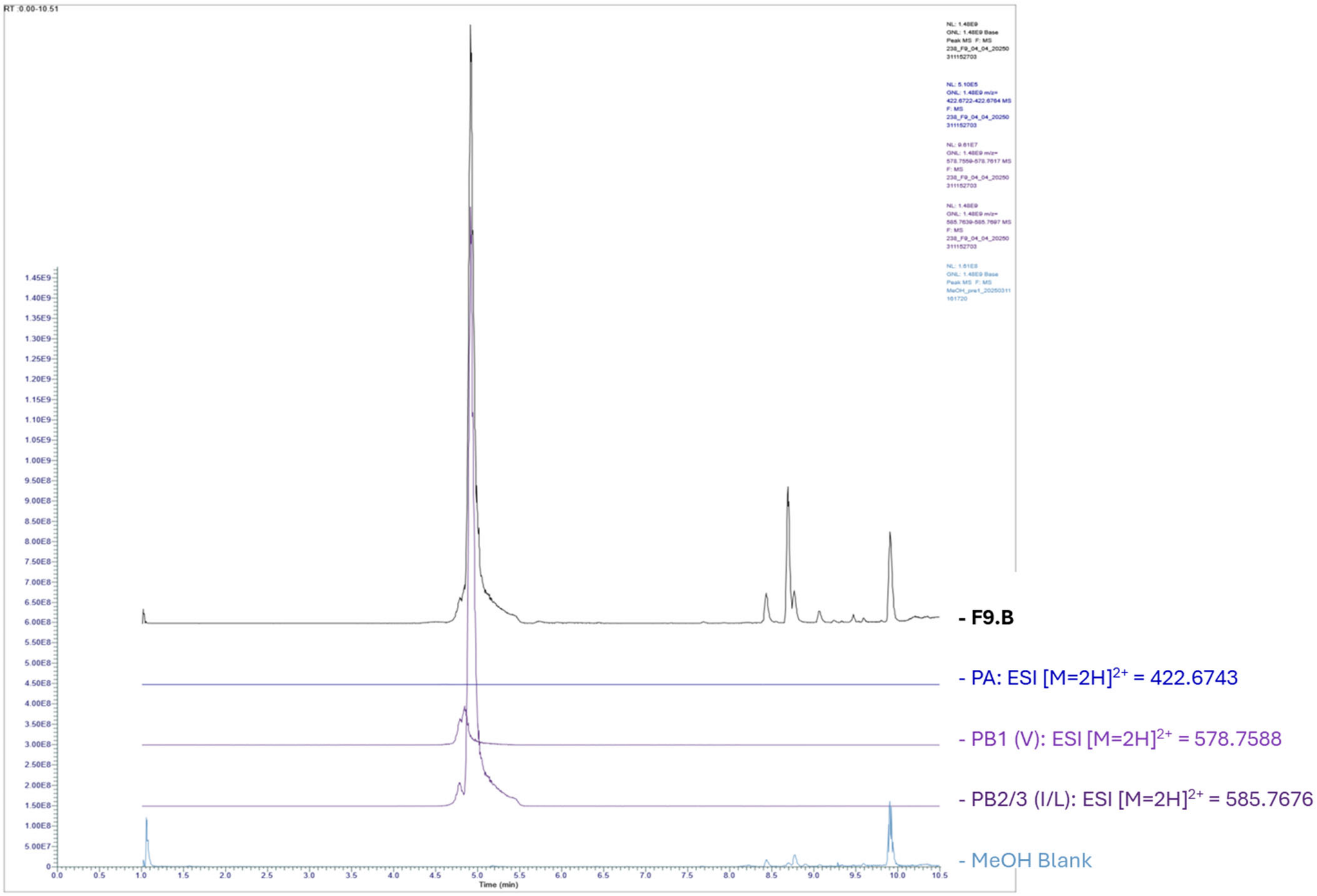


**Figure S8. LCMS data of the HPLC fraction F9.D** The extracted ion chromatograms (EIC) confirmed PB(I/L) as the main component.

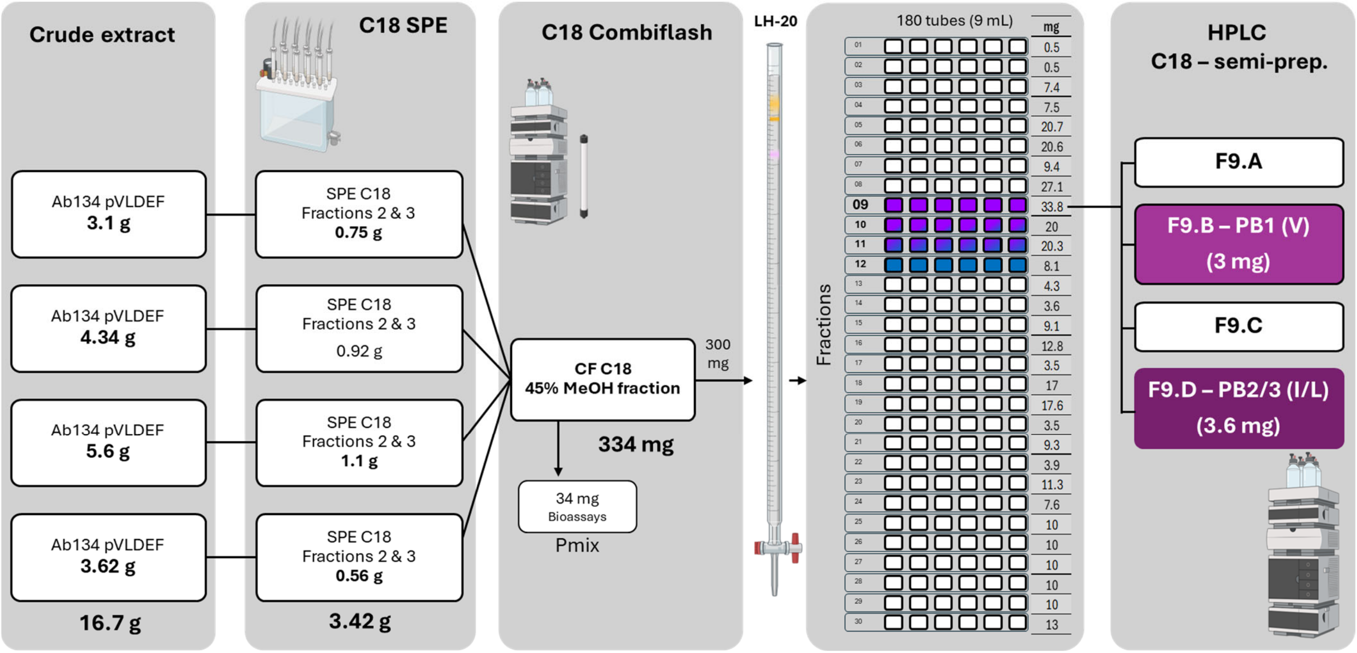


**Figure S9. Workflow of the isolation of pseudovibriamides B1 (V) and B2/3 (I/L).**

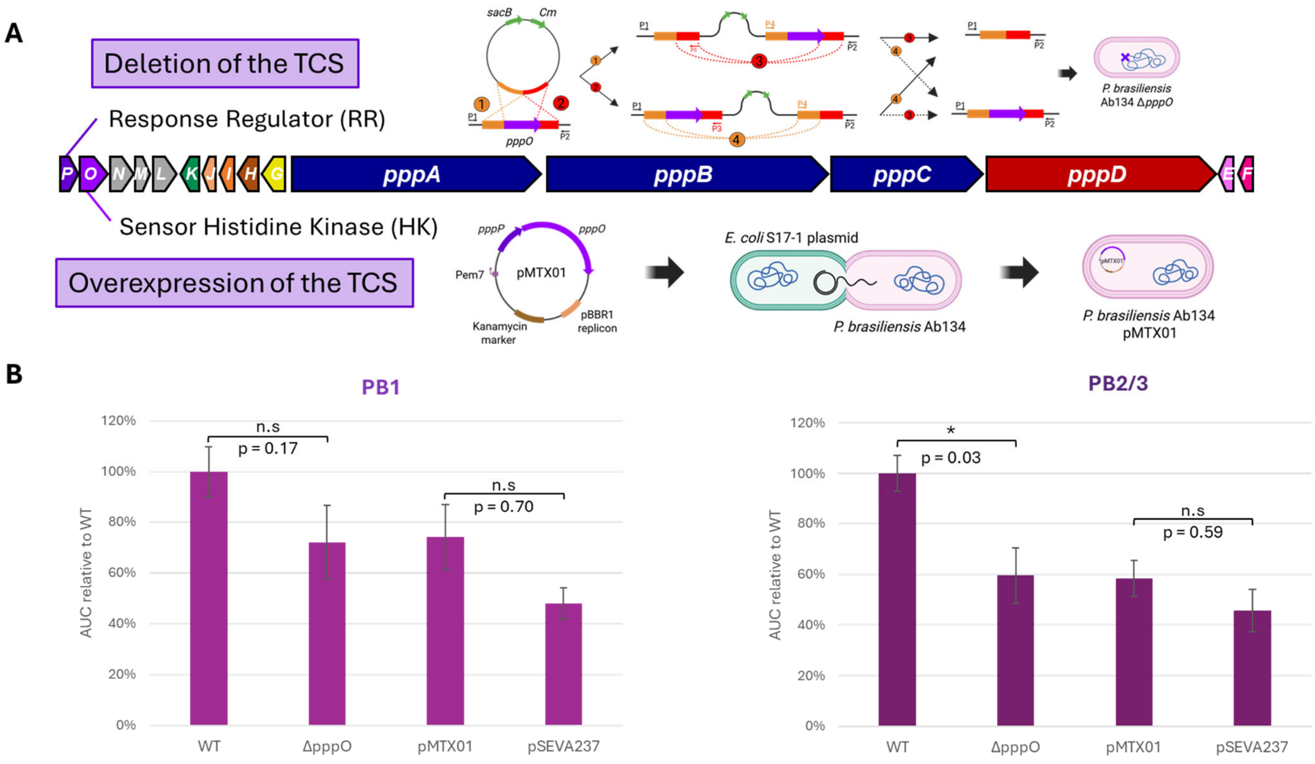


**Figure S10. Testing the involvement of a two-component system in pseudovibriamide biosynthesis.** (**A**) Location of the two-component system (TCS) genes upstream of the *ppp* biosynthetic gene cluster. Method workflow for the in-frame deletion of *pppO* and the overexpression of *pppOP.* (**B**) Relative production of PB1 (V) and PB2/3 (I/L) as determined by LC-MS analysis. *P. brasiliensis* Ab134 wild type strains containing pMTX01 were compared to strains containing the empty vector pSEVA237 that was used to build pMTX01. *P-value < 0.05; n.s., not significant.

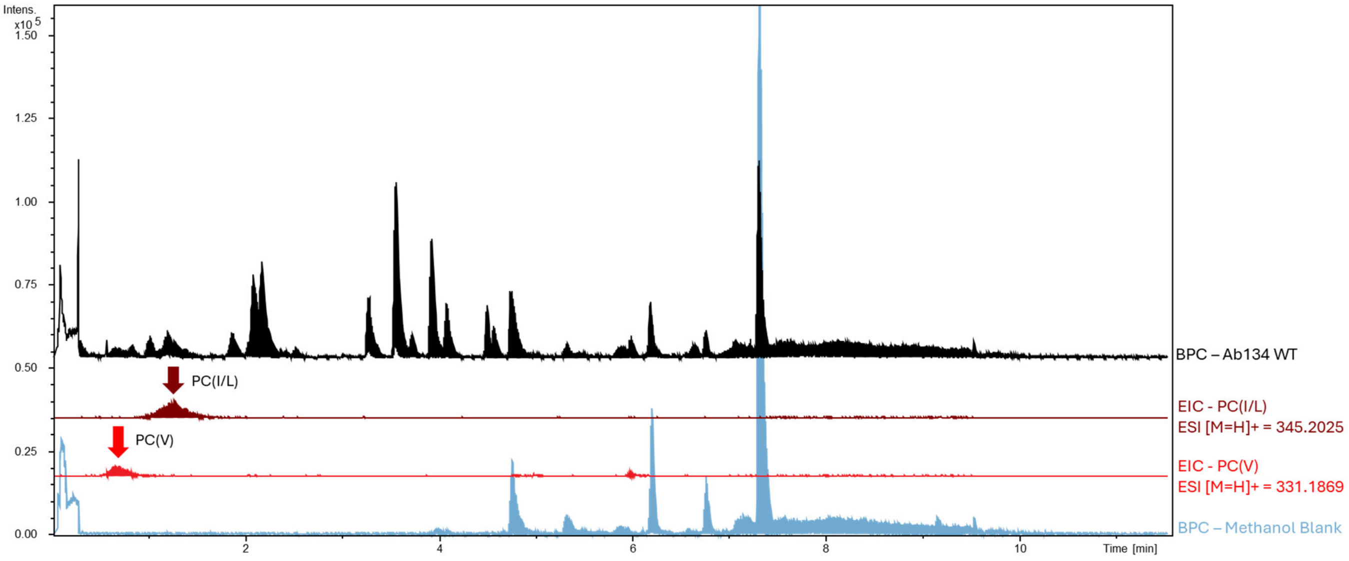


**Figure S11. LCMS data of the crude extract of Ab134 WT.** The extracted ion chromatograms (EIC) show the presence of PC Valine (V) and PC Isoleucine/Leucine (I/L) in the Ab134 WT crude extract analysis as a reference for retention time. The solvent control (methanol) is in blue. The arrows indicate the retention time of PC(V) and PC(I/L) in the EIC chromatograms.

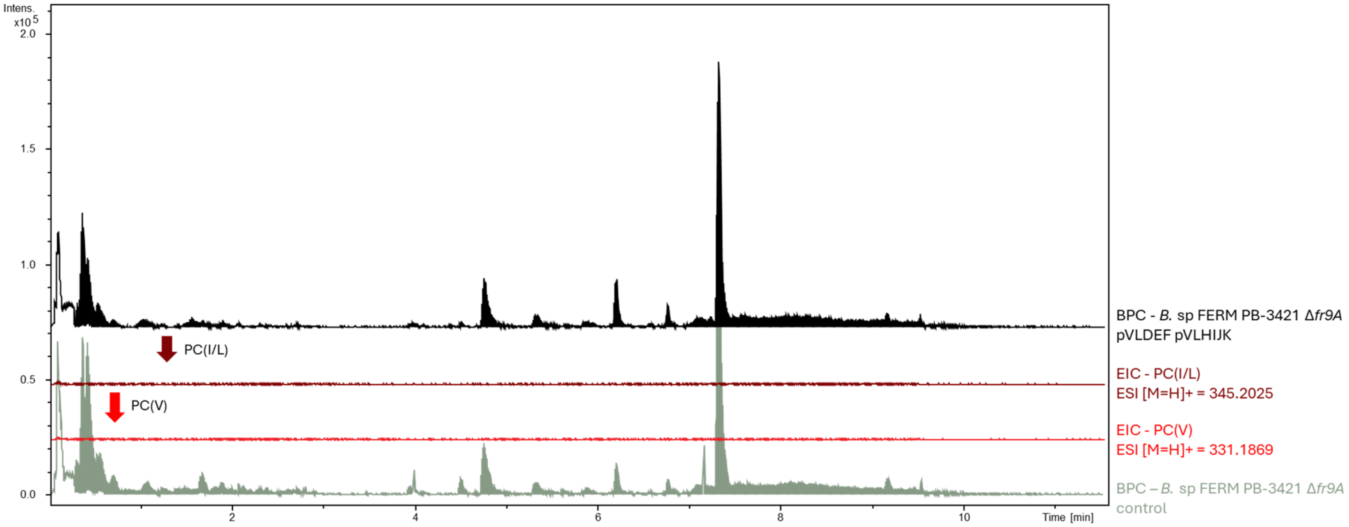


**Figure S12. LCMS data of the crude extract of *Burkholderia* sp FERM PB-3421 ∆*fr9A* pVLDEF pVLHIJK.** The extracted ion chromatograms (EIC) show the absence of PC in the *Burkholderia* extracts. BPC: Base peak chromatogram. The arrows indicate the expected retention time of PB(V) and PB(I/L) in the EIC chromatograms.

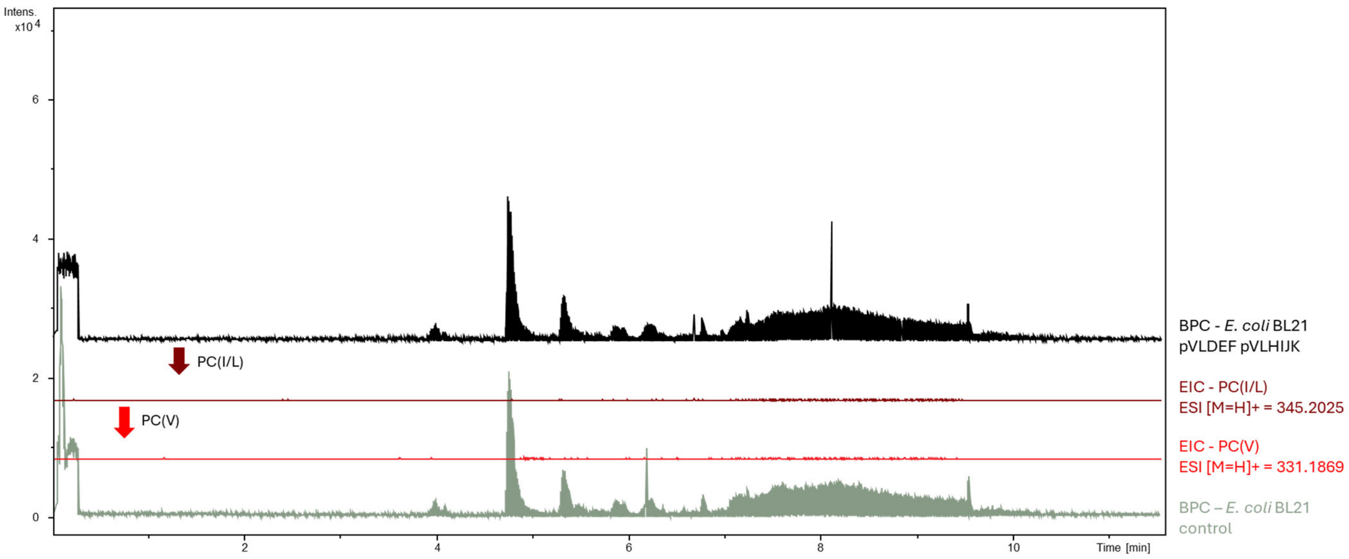


**Figure S13. LCMS data of the crude extract of *E. coli* BL21 pVLDEF pVLHIJK.** The extracted ion chromatograms (EIC) show the absence of PC in *E. coli* extracts. BPC: Base peak. The arrows indicate the expected retention time of PB(V) and PB(I/L) in the EIC chromatograms.

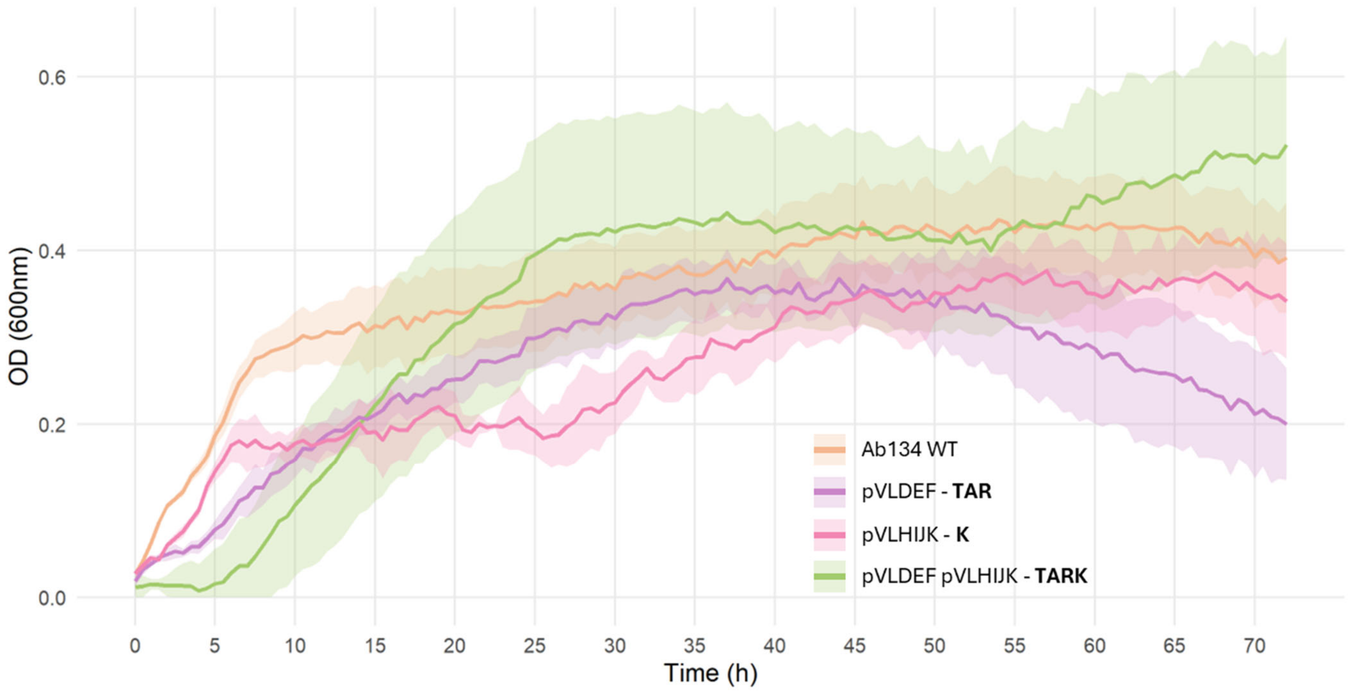


**Figure S14. Growth curves of the four strains depicted in Figure 4A (main text).** Growth was measured based on optical density (OD) at 600 nm. Bold letters after plasmid names represent the antibiotics and promoter inducers used for each culture: Tetracycline (T), kanamycin (K), L-Arabinose (A), and L-Rhamnose (R). Data points were collected every 30 minutes and represent the average of 5 replicates. The shaded bands indicate standard deviation.

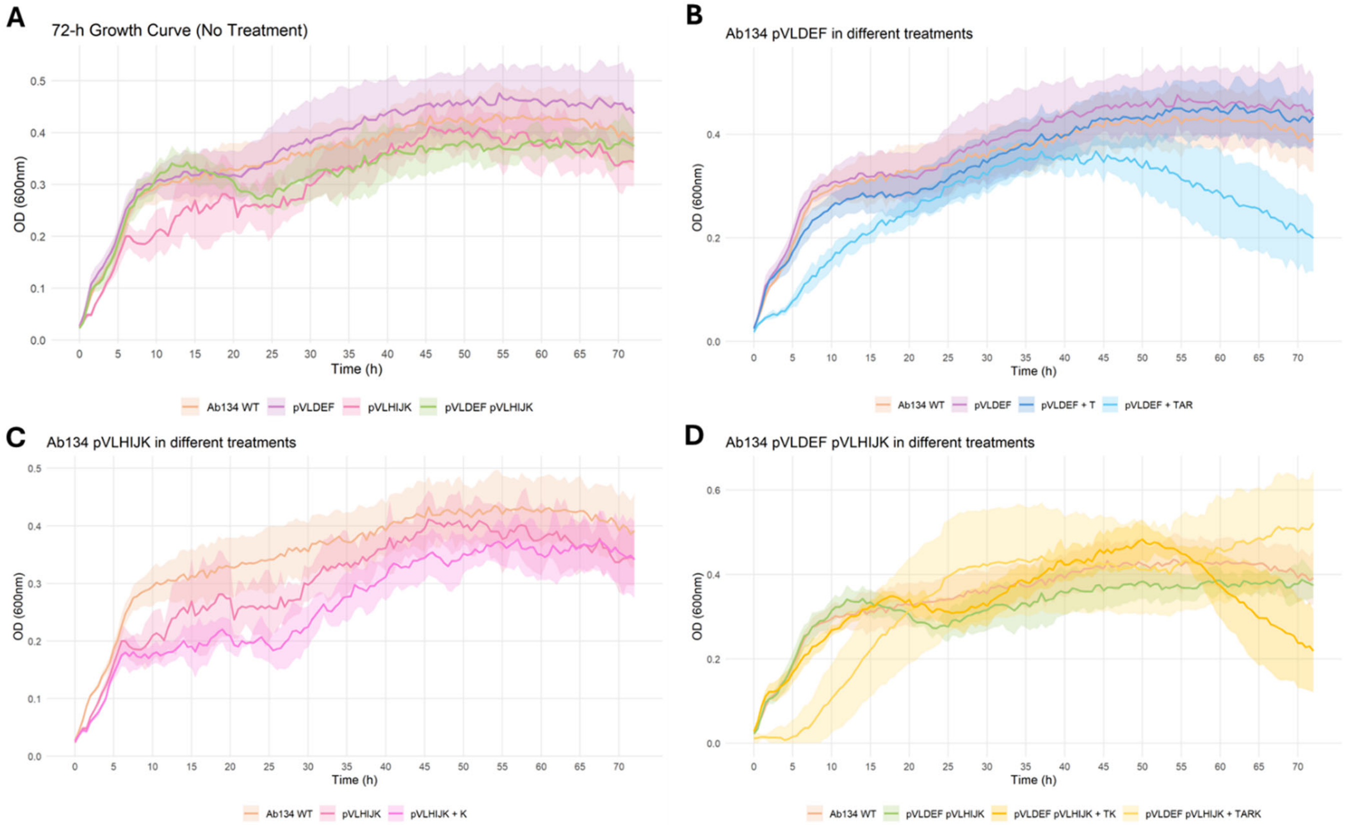


**Figure S15**. **Growth curves of the four strains shown in Figure 4A (main text) under different conditions.** (**A**) All three strains of *P. brasiliensis* with the different plasmids were cultured without any antibiotic or promoter inducers. (**B**) *P. brasiliensis* Ab134 carrying plasmid pVLDEF was cultured with tetracycline alone (T), tetracycline plus promoter inducers L-arabinose and L-rhamnose (TAR), or without antibiotic and inducers. (**C**) *P. brasiliensis* Ab134 carrying plasmid pVLHIJK was cultured with kanamycin (K), or without antibiotics. (**D**) *P. brasiliensis* Ab134 carrying plasmids pVLDEF and pVLHIJK was cultured with tetracycline and kanamycin (TK), tetracycline and kanamycin plus promoter inducers L-arabinose and L-rhamnose (TARK), or without antibiotic and inducers. All growth curves are compared to *P. brasiliensis* Ab134 WT.

A

| BLS concentration (μg/mL) | MAC Pmix 1 (μg/mL) | MAC Pmix 2 (μg/mL) | MAC Pmix 3 (μg/mL) | Mean | Standard Deviation |
|---|---|---|---|---|---|
| 175 | 1 | 1 | 1 | 1.0 | 0.0 |
| 200 | 2 | 2 | 2 | 2.0 | 0.0 |
| 225 | 2 | 2 | 2 | 2.0 | 0.0 |
| 250 | 4 | 4 | 2 | 3.2 | 1.2 |
| 275 | 4 | 4 | 4 | 4.0 | 0.0 |
| 300 | 8 | 8 | 8 | 8 | 0 |
| 325 | 16 | 8 | 8 | 10.1 | 4.6 |
| 350 | 32 | 32 | 64 | 40.3 | 18.5 |
| 375 | - | - | - | - | - |
| 400 | - | - | - | - | - |
| 425 | - | - | - | - | - |
| 450 | - | - | - | - | - |

B

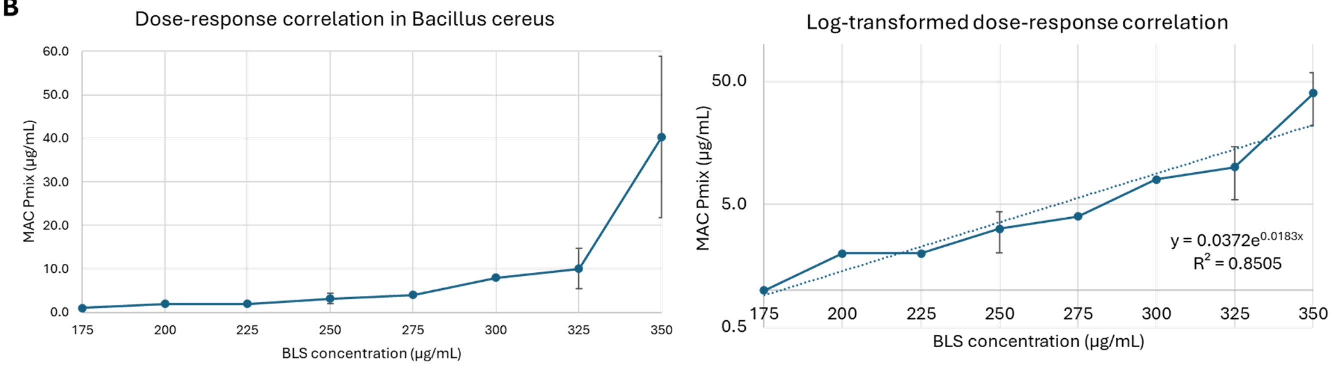


**Figure S16. Dose-response correlation of the antidote activity under increasing concentrations of blasticidin S against *Bacillus cereus* NRRL B-3711**. (**A**) Concentrations of blasticidin S tested and corresponding minimum antidote concentrations (MAC) of Pmix required for bacterial growth recovery. Data are presented as the mean and standard deviation of three biological replicates. (**B**) Dose–response curve showing the increase in MAC Pmix as a function of blasticidin S (BLS) concentration. (**C**) Log-transformed plot of the same data indicating an exponential relationship between BLS concentration and MAC Pmix ($y = 0.0372e^{0.0183x}, R^2 = 0.8505$).

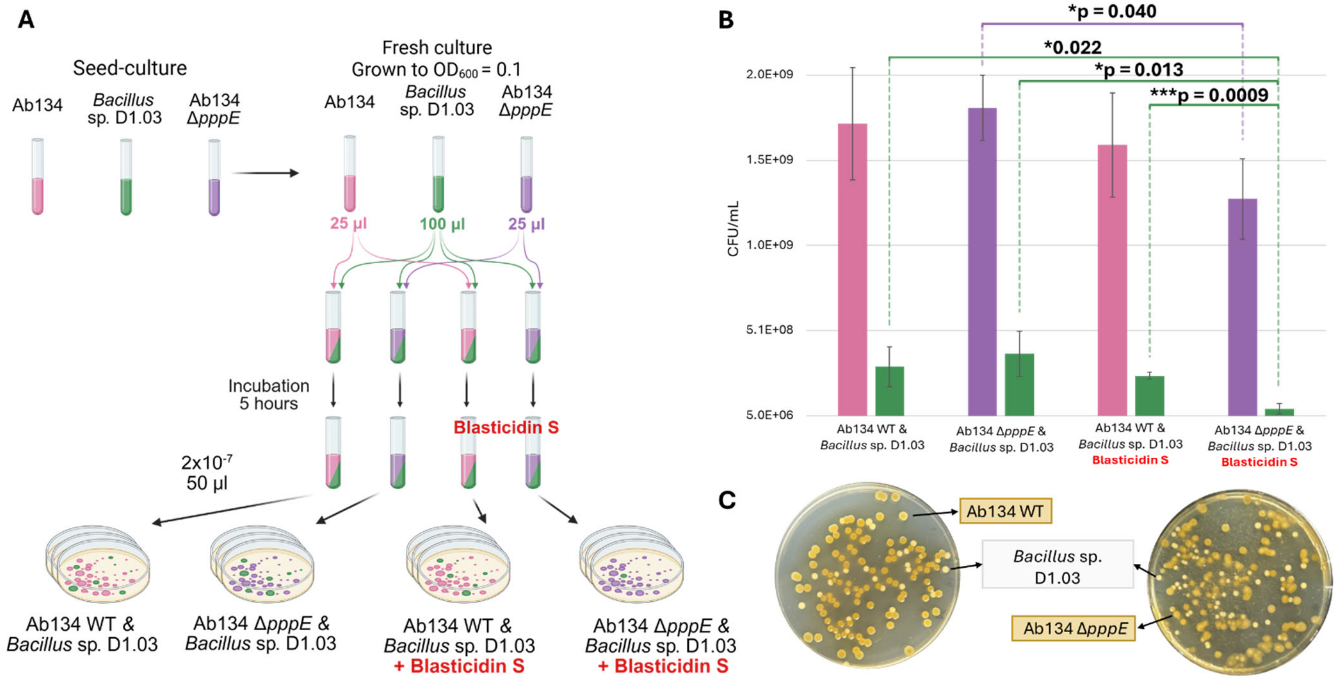


**Figure S17. Co-culture assay of the protected strain *Bacillus* sp. D1.03 and *P. brasiliensis* Ab134 (WT and ∆*pppE*).** (**A**) Method workflow of the co-culture assay. (**B**) CFU/mL counting for each strain present in all 4 conditions of co-culture tested. Statistical significance was determined using a unpaired two-tailed t-test for each pairwise comparison (n = 3 CFU counting plates per condition) * 0.05 < P-value, *** P-value < 0.01. (**C**) Representative co-culture plates used for counting single colonies. Colonies of *P. brasiliensis* Ab134 WT and ∆*pppE* (brownish yellow) are morphologically different from the single colonies of *Bacillus* sp. D1.03 (smaller, white colonies). Under blasticidin S there was a 29% reduction in the number of ∆*pppE* colonies, whereas the reduction in *Bacillus* sp. D1.03 colonies was 86%.

## References

1. Mangalea MR, Borlee BR. The NarX-NarL two-component system regulates biofilm formation, natural product biosynthesis, and host-associated survival in *Burkholderia pseudomallei*. *Sci Rep* 2022;12. https://doi.org/10.1038/s41598-021-04053-6

2. Silva-Rocha R, Martínez-García E, Calles B, et al. The Standard European Vector Architecture (SEVA): A coherent platform for the analysis and deployment of complex prokaryotic phenotypes. *Nucleic Acids Res* 2013;41:666–675. https://doi.org/10.1093/nar/gks1119

3. Fernandez HN, Kretsch AM, Kunakom S, Kadjo AE, Mitchell DA, Eustáquio AS. High-Yield Lasso Peptide Production in a *Burkholderia* Bacterial Host by Plasmid Copy Number Engineering. *ACS Synth Biol* 2024;13:337–350. https://doi.org/10.1021/acssynbio.3c00597

4. Jeske M, Altenbuchner J. The *Escherichia coli* rhamnose promoter rhaP$_{BAD}$ is in *Pseudomonas putida* KT2440 independent of Crp-cAMP activation. *Appl Microbiol Biotechnol* 2010;85:1923–1933. https://doi.org/10.1007/s00253-009-2245-8

5. Dai Y, Lourenzon V, Ióca LP, et al. Pseudovibriamides from *Pseudovibrio* marine sponge bacteria promote flagellar motility via transcriptional modulation. *mBio* 2024;16.

6. Rua CPJ, Trindade-Silva AE, Appolinario LR, et al. Diversity and antimicrobial potential of culturable heterotrophic bacteria associated with the endemic marine sponge *Arenosclera brasiliensis*. *PeerJ* 2014;2014:1–14. https://doi.org/10.7717/peerj.419

7. Frankland GC, Frankland PF. Studies on some new microorganisms obtained from air. 1887. https://doi.org/10.1098/rstb.1887.0011/1274102/rstb.1887.0011.pdf

8. Webster NS, Negri AP, Webb RI, Hill RT. A spongin-boring α-proteobacterium is the etiological agent of disease in the Great Barrier Reef sponge *Rhopaloeides odorabile*. *Mar Ecol Prog Ser* 2002;232:305–309. https://doi.org/https://doi.org/10.3354/meps232305

9. Ben-Haim Y, Thompson FL, Thompson CC, et al. *Vibrio coralliilyticus* sp. nov., a temperature-dependent pathogen of the coral *Pocillopora damicornis*. *Int J Syst Evol Microbiol* 2003;53:309–315. https://doi.org/10.1099/ijs.0.02402-0

10. Versluis D, McPherson K, van Passel MWJ, Smidt H, Sipkema D. Recovery of Previously Uncultured Bacterial Genera from Three Mediterranean Sponges. *Marine Biotechnology* 2017;19:454–468. https://doi.org/10.1007/s10126-017-9766-4

11. Indraningrat AAG, Micheller S, Runderkamp M, et al. Cultivation of Sponge-Associated Bacteria from *Agelas sventres* and *Xestospongia muta* Collected from Different Depths. *Mar Drugs* 2019;17. https://doi.org/10.3390/md17100578

12. Paul SI, Rahman MM, Salam MA, Khan MAR, Islam MT. Identification of marine sponge-associated bacteria of the Saint Martin's island of the Bay of Bengal emphasizing on the prevention of motile *Aeromonas septicemia* in *Labeo rohita*. *Aquaculture* 2021;545. https://doi.org/10.1016/j.aquaculture.2021.737156

13. Suzumura K et al. YM-266183 and YM-266184, Novel thiopeptide antibiotics produced by *Bacillus cereus* isolated from a marine sponge. *J Antibiot (Tokyo)* 2003;56:129–134. doi: 10.7164/antibiotics.56.129
